# Genetic Architecture of Abdominal Aortic Aneurysm in the Million Veteran Program

**DOI:** 10.1161/CIRCULATIONAHA.120.047544

**Published:** 2020-09-28

**Authors:** Derek Klarin, Shefali Setia Verma, Renae Judy, Ozan Dikilitas, Brooke N. Wolford, Ishan Paranjpe, Michael G. Levin, Cuiping Pan, Catherine Tcheandjieu, Joshua M. Spin, Julie Lynch, Themistocles L. Assimes, Linn Åldstedt Nyrønning, Erney Mattsson, Todd L. Edwards, Josh Denny, Eric Larson, Ming Ta Michael Lee, David Carrell, Yanfei Zhang, Gail P. Jarvik, Ali G. Gharavi, John Harley, Frank Mentch, Jennifer A. Pacheco, Hakon Hakonarson, Anne Heidi Skogholt, Laurent Thomas, Maiken Elvestad Gabrielsen, Kristian Hveem, Jonas Bille Nielsen, Wei Zhou, Lars Fritsche, Jie Huang, Pradeep Natarajan, Yan V. Sun, Scott L. DuVall, Daniel J. Rader, Kelly Cho, Kyong-Mi Chang, Peter W.F. Wilson, Christopher J. O’Donnell, Sekar Kathiresan, Salvatore T. Scali, Scott A. Berceli, Cristen Willer, Gregory T. Jones, Matthew J. Bown, Girish Nadkarni, Iftikhar J. Kullo, Marylyn Ritchie, Scott M. Damrauer, Philip S. Tsao

**Affiliations:** 1 Malcolm Randall VA Medical Center, Gainesville, FL (D.K., S.T.S., S.A.B.).; 2 Division of Vascular Surgery and Endovascular Therapy, University of Florida College of Medicine, Gainesville (D.K., S.T.S., S.A.B.).; 3 Center for Genomic Medicine (D.K., W.Z., P.N.), Massachusetts General Hospital, Harvard Medical School, Boston.; 4 Department of Medicine (P.N.), Massachusetts General Hospital, Harvard Medical School, Boston.; 5 Program in Medical and Population Genetics (D.K.), Broad Institute of MIT and Harvard, Cambridge, MA.; 6 Stanley Center for Psychiatric Research (W.Z.), Broad Institute of MIT and Harvard, Cambridge, MA.; 7 Department of Genetics (S.S.V., M.R.), Perelman School of Medicine, University of Pennsylvania, Philadelphia.; 8 Department of Surgery (R.J., S.M.D.), Perelman School of Medicine, University of Pennsylvania, Philadelphia.; 9 Division of Cardiovascular Medicine (M.G.L.), Perelman School of Medicine, University of Pennsylvania, Philadelphia.; 10 Department of Medicine (M.G.L., D.J.R., K.-M.C.), Perelman School of Medicine, University of Pennsylvania, Philadelphia.; 11 Department of Pediatrics (H.H.), Perelman School of Medicine, University of Pennsylvania, Philadelphia.; 12 Corporal Michael J. Crescenz VA Medical Center, Philadelphia, PA (R.J., M.G.L., K.-M.C., S.M.D.).; 13 Department of Cardiovascular Medicine, Mayo Clinic, Rochester, MN (O.D., I.J.K.).; 14 Department of Computational Medicine and Bioinformatics (B.N.W., C.W.), University of Michigan Medical School, Ann Arbor.; 15 Department of Biostatistics (L.F.), University of Michigan Medical School, Ann Arbor.; 16 Department of Internal Medicine, Division of Cardiology (C.W.), University of Michigan Medical School, Ann Arbor.; 17 Department of Human Genetics (C.W.), University of Michigan Medical School, Ann Arbor.; 18 Department of Medicine, Icahn School of Medicine at Mount Sinai, New York, NY (I.P., G.N.).; 19 Palo Alto Epidemiology Research and Information Center for Genomics (C.P.), CA.; 20 VA Palo Alto Health Care System (C.T., J.M.S., T.L.A., P.S.T.), CA.; 21 Division of Cardiovascular Medicine, Department of Medicine (C.T., J.M.S., T.L.A., P.S.T.), Stanford University School of Medicine, CA.; 22 Department of Pediatric Cardiology (C.T.), Stanford University School of Medicine, CA.; 23 Edith Nourse VA Medical Center, Bedford, MA (J.L.).; 24 VA Informatics and Computing Infrastructure, VA Salt Lake City Health Care System, UT (J.L., S.L.D.).; 25 Department of Vascular Surgery, St. Olavs Hospital, Trondheim, Norway (L.Å.N., E.M.).; 26 Department of Circulation and Medical Imaging (L.Å.N., E.M.), Norwegian University of Science and Technology, Trondheim, Norway.; 27 Faculty of Medicine and Health Sciences (A.H.S., L.T., M.E.G., K.H., J.B.N.), Norwegian University of Science and Technology, Trondheim, Norway.; 28 Department of Clinical and Molecular Medicine (L.T.), Norwegian University of Science and Technology, Trondheim, Norway.; 29 Division of Epidemiology, Department of Medicine, Vanderbilt-Ingram Cancer Center (T.L.E.), Vanderbilt University Medical Center, Nashville, TN.; 30 Vanderbilt Genetics Institute (T.L.E., J.D.), Vanderbilt University Medical Center, Nashville, TN.; 31 Department of Biomedical Informatics (J.D., E.L., D.C.), Vanderbilt University Medical Center, Nashville, TN.; 32 Kaiser Permanente Washington Health Research Institute, Seattle (J.D., E.L., D.C.).; 33 Departments of Medicine and Health Services (E.L.), University of Washington, Seattle.; 34 Division of Medical Genetics, Departments of Medicine and Genome Sciences (G.P.J.), University of Washington, Seattle.; 35 Genomic Medicine Institute, Geisinger Health System, Danville, PA (M.T.M.L., Y.Z.).; 36 Division of Nephrology and Center for Precision Medicine and Genomics, Columbia University, New York, NY (A.G.G.).; 37 Center for Autoimmune Genomics and Etiology (CAGE), Cincinnati Children’s Hospital Medical Center, OH (J.H.).; 38 Department of Pediatrics, University of Cincinnati College of Medicine, OH (J.H.).; 39 US Department of Veterans Affairs, Cincinnati, OH (J.H.).; 40 Center for Applied Genomics, The Children’s Hospital of Philadelphia, PA (F.M., H.H.).; 41 Center for Genetic Medicine, Northwestern University Feinberg School of Medicine, Chicago, IL (J.A.P.).; 42 K.G. Jebsen Center for Genetic Epidemiology, Department of Public Health and Nursing, Department of Epidemiology Research, Statens Serum Institute, Copenhagen, Denmark (J.B.N.).; 43 Analytic and Translational Genetics Unit (W.Z.), Massachusetts General Hospital, Boston.; 44 Cardiovascular Research Center (P.N.), Massachusetts General Hospital, Boston.; 45 Boston VA Healthcare System, MA (J.H., P.N., K.C., C.J.O.).; 46 Department of Epidemiology, Emory University Rollins School of Public Health, Atlanta, GA (Y.V.S.).; 47 Atlanta VA Health Care System, Decatur, GA (Y.V.S., P.W.F.W.).; 48 Division of Epidemiology, Department of Internal Medicine, University of Utah School of Medicine, Salt Lake City (S.L.D.).; 49 Emory Clinical Cardiovascular Research Institute, Atlanta, GA (P.W.F.W.).; 50 Cardiovascular Medicine Division, Department of Medicine, Brigham and Women’s Hospital, Harvard Medical School, Boston, MA (C.J.O.).; 51 Verve Therapeutics, Cambridge, MA (S.K.).; 52 Department of Surgical Sciences, Dunedin School of Medicine, University of Otago, New Zealand (G.T.J.).; 53 Department of Cardiovascular Sciences and NIHR Leicester Biomedical Research Centre, University of Leicester, United Kingdom (M.J.B.).

**Keywords:** aneurysm, aortic diseases, genome-wide association study, humans

## Abstract

Abdominal aortic aneurysm (AAA) is an important cause of cardiovascular mortality; however, its genetic determinants remain incompletely defined. In total, 10 previously identified risk loci explain a small fraction of AAA heritability.

**Methods:**

We performed a genome-wide association study in the Million Veteran Program testing ≈18 million DNA sequence variants with AAA (7642 cases and 172 172 controls) in veterans of European ancestry with independent replication in up to 4972 cases and 99 858 controls. We then used mendelian randomization to examine the causal effects of blood pressure on AAA. We examined the association of AAA risk variants with aneurysms in the lower extremity, cerebral, and iliac arterial beds, and derived a genome-wide polygenic risk score (PRS) to identify a subset of the population at greater risk for disease.

**Results:**

Through a genome-wide association study, we identified 14 novel loci, bringing the total number of known significant AAA loci to 24. In our mendelian randomization analysis, we demonstrate that a genetic increase of 10 mm Hg in diastolic blood pressure (odds ratio, 1.43 [95% CI, 1.24–1.66]; *P*=1.6×10^−6^), as opposed to systolic blood pressure (odds ratio, 1.06 [95% CI, 0.97–1.15]; *P*=0.2), likely has a causal relationship with AAA development. We observed that 19 of 24 AAA risk variants associate with aneurysms in at least 1 other vascular territory. A 29-variant PRS was strongly associated with AAA (odds ratio_PRS_, 1.26 [95% CI, 1.18–1.36]; *P*_PRS_=2.7×10^−11^ per SD increase in PRS), independent of family history and smoking risk factors (odds ratio_PRS+family history+smoking_, 1.24 [95% CI, 1.14–1.35]; *P*_PRS_=1.27×10^−6^). Using this PRS, we identified a subset of the population with AAA prevalence greater than that observed in screening trials informing current guidelines.

**Conclusions:**

We identify novel AAA genetic associations with therapeutic implications and identify a subset of the population at significantly increased genetic risk of AAA independent of family history. Our data suggest that extending current screening guidelines to include testing to identify those with high polygenic AAA risk, once the cost of genotyping becomes comparable with that of screening ultrasound, would significantly increase the yield of current screening at reasonable cost.

## Abstract 2

Supplemental Digital Content is available in the text.

**Clinical Perspective**

**What Is New?**

- A genome-wide association study of abdominal aortic aneurysm (AAA) further defines the role of common genetic variation in disease susceptibility.
- Mendelian randomization analysis supports a causal role for smoking liability and diastolic blood pressure in the pathogenesis of AAA.
- A polygenic risk score based on 29 genome-wide AAA risk variants strongly associates with AAA independent of family history and 6 additional clinical risk factors.

**What Are the Clinical Implications?**

- These results highlight novel AAA genetic associations with therapeutic implications, including *LPA* and *PCSK9.*
- Diastolic blood pressure, as opposed to systolic blood pressure, is likely of greater significance in the pathogenesis of AAA.
- Polygenic risk score analysis can identify a subset of the population at significantly increased genetic risk of AAA independent of family history; extending current screening guidelines to include testing for those with high polygenic AAA risk deserves further investigation, particularly once genotyping becomes standard of care in healthcare systems and can be performed at nominal cost.

Abdominal aortic aneurysm (AAA) is a complex disease affected by both environmental^1^ and genetic factors,^2^ and heritability has been estimated to be as high as 70%.^3^ Genome-wide association studies (GWAS) have revealed only 10 loci reaching genome-wide significance,^4–7^ leaving a significant portion of AAA heritability unknown. Previously published discovery efforts have been limited by small sample sizes, with the largest previous GWAS analyzing ≈5000 AAA cases.^7^

Large-scale biobanks combining genetic data with electronic health record (EHR)–derived phenotypes have continued to evolve over the past decade.^8,9^ The Million Veteran Program (MVP) was established in 2011 to study how genes affect health in the Veterans Health Administration. Recent efforts have demonstrated that a Veterans Health Administration–based biobank provides the ability to aid genetic discovery for understudied vascular diseases with a higher prevalence among US veterans.^10,11^ Leveraging the MVP resource, we sought to perform a genetic discovery analysis for AAA; explore the spectrum of phenotypic consequences associated with AAA risk variants; examine the genetic relationship between smoking and blood pressure and AAA; test the effects of AAA risk variants on aneurysms in other territories; and develop and evaluate a genome-wide polygenic risk score (PRS) for AAA to identify a subset of the population at higher risk for disease.

## Methods

The full summary-level association data from the MVP AAA discovery analysis from this article are available through dbGaP (the database of Genotypes and Phenotypes; accession code: phs001672.v2.p1).

### Study Populations

**Figure 1.**
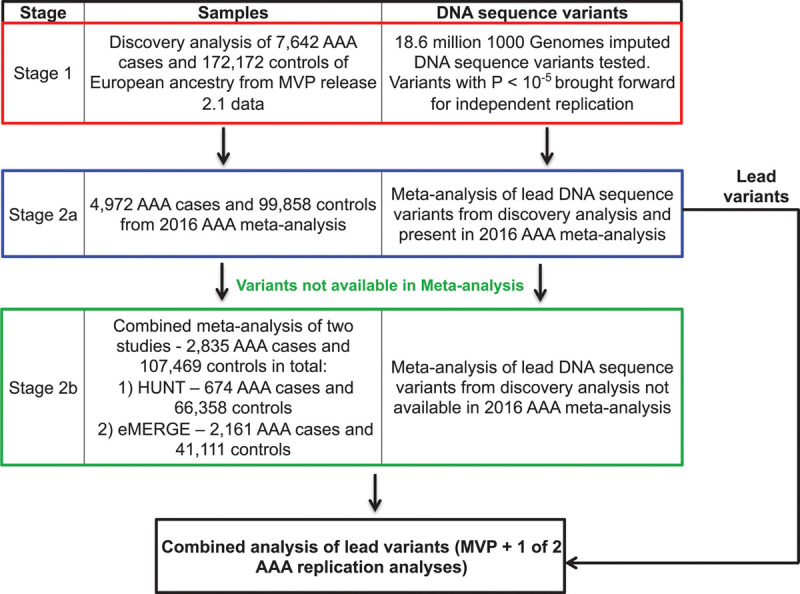
**Abdominal aortic aneurysm (AAA) genome-wide association study design.** DNA sequence variants with suggestive association (*P*<10^−5^) in discovery (stage 1) were brought forward for independent replication and tested using summary statistics from the 2016 AAA meta-analysis consisting of 4972 AAA cases and 99 858 controls^7^ (stage 2a) or from a combined meta-analysis of HUNT (the Trøndelag *Health* Study) and the eMERGE Network (electronic Medical Records and Genomics) datasets consisting of a total of 2835 AAA cases and 107 469 controls (stage 2b) if variants were not available in stage 2a. MVP indicates Million Veteran Program.

We conducted a discovery genetic association analysis using DNA samples and phenotypic data from the MVP (Figure 1 and Figure I in the Data Supplement). In the MVP, individuals ages 18 to >100 years have been recruited from 63 Veterans Affairs medical centers across the United States. After performing quality control, we identified 227 817 European participants for AAA discovery analysis from MVP release 2.1. After applying our phenotypic algorithm, we evaluated 7642 AAA cases and 172 172 controls free from clinical evidence of disease. For variants with suggestive associations (*P*<10^−5^), we sought replication of our findings with data from either of 2 independent datasets. We first attempted replication of lead AAA variants using summary statistics from the 2016 AAA meta-analysis consisting of 4972 AAA cases and 99 858 controls^7^ (stage 2a).

If association statistics for promising DNA sequence variants from stage 1 were not available for replication, we sought replication of these variants from a combined meta-analysis of 2 studies: HUNT (the Trøndelag *Health* Study; 674 AAA cases and 66 358 controls) and the eMERGE Network (electronic Medical Records and Genomics; 2161 AAA cases and 41 111 controls). Network datasets consisted of up to 2835 AAA cases and 107 469 controls in total (stage 2b). HUNT is a population-based health survey conducted in the county of Nord-Trøndelag, Norway, since 1984. Individuals were included at 3 different time points over ≈20 years (HUNT1 [1984 to 1986], HUNT2 [1995 to 1997], and HUNT3 [2006 to 2008]). The eMERGE Network is a collaboration of genetic biobanks with phenotypic data linked to EHRs. The study design and sample collection of eMERGE Network cohorts has been described previously.^12^ Deidentified samples linked to EHRs were supplied from multiple investigators from different institutions with approval across the consortium for analysis. Additional details of genetic data and quality control are available in the Data Supplement.

The MVP received ethical and study protocol approval by the Veterans Affairs Central Institutional Review Board and informed consent was obtained for all participants. Each additional study received approval from its local institutional review board.

### AAA Phenotype Definitions

From the participants passing quality control in MVP, individuals were defined as having AAA or being a disease-free control using a previously adjudicated^13^ definition initially proposed by Denny et al.^14^ AAA cases were defined as the presence of 2 instances of any of the following International Classification of Diseases (ICD)–9 or ICD-10 codes in a participant’s EHR: 441.3, 441.4, I71.3, or I71.4. Controls were defined as possessing no occurrences of the aforementioned ICD codes, as well as no occurrences of the ICD-9 codes 440 through 448 or ICD-10 codes I71 through I75, I77 through I79, or K55. This identified 7642 AAA cases and 172 172 controls of European ancestry for genetic discovery, and an independent set of 1656 AAA cases and 44 908 controls of European ancestry and 718 AAA cases and 46 380 controls of African ancestry for our PRS analysis. In HUNT, all patients diagnosed with AAA from 1995 through 2014 in Nord-Trøndelag were identified from hospital and outpatient registries from the 3 local hospitals: St Olav’s Hospital, Levanger Hospital, and Namsos Hospital. All diagnoses were verified manually using patient chart data. The unique personal identification number was used to link data on AAA diagnoses and death with exposure data from HUNT. This identified 674 AAA cases and 66 358 controls for downstream analysis. In the eMERGE Network, BioMe, and the Penn Medicine Biobank, AAA was defined in a manner identical to the definition proposed by Denny et al.^14^ In the Mayo Clinic Vascular Disease Biorepository, which consists of patients referred for noninvasive vascular evaluation including abdominal ultrasound, AAA cases were defined as having an infrarenal abdominal aortic diameter ≥3 cm or a history of open or endovascular AAA repair. Controls were not known to have AAA and had no ICD-9 diagnosis codes for AAA.

### Phenome-Wide Association Study of AAA Risk Variants

Of the genotyped veterans passing quality control, we identified 23 172 451 distinct, prevalent ICD diagnosis codes available for analysis. We focused on the entire group of 227 817 White participants passing quality control, in which the mean age was 64.3±13.1 years, and 93.3% (212 465) were male. ICD-9 or ICD-10 diagnosis codes were collapsed to clinical disease groups and corresponding controls using the groupings proposed by Denny et al^14^ and tested using logistic regression. Diseases were required to have a prevalence of >0.13% (≈300 cases) to be included in the Phenome-Wide Association Study (PheWAS) analysis. We tested a total of 1249 distinct diseases, symptoms, and injuries in MVP using the R package PheWAS.^15^

### Shared Heritability With AAA Risk Factors

To better understand how common genetic variation influences risk for AAA and its risk factors, we used linkage disequilibrium score regression^16^ to calculate the genetic correlation between AAA and 10 known risk factors for disease and aneurysm rupture. MVP association summary statistics for AAA were uploaded to LD Hub (http://ldsc.broadinstitute.org/ldhub/) and genetic correlation analyses performed between AAA, 4 smoking categories (current smoker, ever smoker, cigarettes/day, and chronic obstructive pulmonary disease), and 2 blood pressure traits (systolic blood pressure [SBP] and diastolic blood pressure [DBP]) from UK Biobank and previously published GLGC plasma lipid (low-density lipoprotein cholesterol, high-density lipoprotein cholesterol, and triglycerides)^17^ and coronary artery disease statistics from the CARDIoGRAMplusC4D (Coronary Artery Disease Genome-Wide Replication and Meta-Analysis Plus Coronary Artery Disease Genetics) GWAS.^18^

### Smoking and Blood Pressure Mendelian Randomization Analyses

Mendelian randomization (MR) analyses for 3 smoking phenotypes (smoking initiation, smoking heaviness, and smoking cessation) and 2 blood pressure phenotypes (SBP and DBP) with AAA development were performed. Genetic instruments were selected as DNA sequence variants that associated with the exposure at genome-wide significance (*P*<5×10^−8^) with an *R*^2^<0.001. All clumping was performed using the TwoSampleMR package of R.^19^ Genetic instruments for the smoking initiation, smoking heaviness, and smoking cessation exposures were derived from publicly available GSCAN (GWAS & Sequencing Consortium of Alcohol and Nicotine Use) data from up to 1 232 091 participants^20^; instruments of the SBP and DBP traits were derived from the International Consortium of Blood Pressure and UK Biobank Discovery GWAS^21^ analysis in up to 757 601 individuals. Inverse variance–weighted MR was used for the primary analysis, with weighted median^22^ MR performed as sensitivity analysis, allowing for up to 50% of the weight of each instrument to be drawn from invalid instruments while controlling type I error. Diagnostic leave-one-out, single-variant, funnel-plot, and MR-Egger^23^ analyses were performed to evaluate for evidence of heterogeneity and horizontal pleiotropy. In addition, we performed the MR-PRESSO test (Mendelian Randomization Pleiotropy Residual Sum and Outlier)^24^ to identify evidence of horizontal pleiotropy, which consists of 3 parts: (1) the global test for horizontal pleiotropy; (2) the outlier corrected causal estimate, which corrects for the detected horizontal pleiotropy; and (3) the distortion test, which tests whether the causal estimate is significantly different after outlier adjustment.

### Aneurysm Associations in Other Vascular Territories

We sought to better understand how DNA sequence variants might differ in contribution to risk for aneurysmal disease in the iliac, lower extremity, and cerebral vascular beds. We first examined the strength of the relationship between AAA and aneurysms in other vascular beds. Next, for lead AAA risk variants identified in our primary analysis, we tested their effect on iliac, lower extremity, and cerebral aneurysms in European MVP participants using the definition proposed by Denny et al.^14^ We performed a sensitivity analysis to examine the association with isolated aneurysms in these territories by excluding individuals with a diagnosis of AAA from the analysis.

### AAA PRS Generation

A weighted PRS represents an individual’s risk of a given disease conferred by the sum of the effects of many common DNA sequence variants. A weight is assigned to each genetic variant based on its strength of association with disease risk (β). Individuals are then additively scored in a weighted fashion based on the number of risk alleles they carry for each variant in the PRS.

To generate our scores, we used summary statistics from the MVP release 2.1 European ancestry GWAS analysis (7642 AAA cases, 172 172 controls) and a linkage disequilibrium panel of 20 000 randomly selected European samples from UK Biobank. To increase the number of independent variants included in our score, we performed a pruning and thresholding analysis using the linkage disequilibrium–driven clumping procedure in PLINK version 1.90b (clump). In brief, this algorithm formed “clumps” around variants with AAA association (in this case, *P*<0.01, *P*<1×10^−4^, *P*<1×10^−6^) and with an *R*^2^>0.0001 based on the linkage disequilibrium reference. From our initial set of summary statistics, the algorithm selected only 1 associated variant from each clump below our prespecified *P* value threshold. The final output from this procedure generated 3 scores of 29, 301, and 3699 independent (*R*^2^<0.0001) AAA-associated variants, representing the strongest disease-associated variant for each linkage disequilibrium–based clump across the genome.

### AAA PRS Analysis

We first tested the associated AAA risk for each of the PRS_AAA_ using ascertained AAA case–control cohort data from the Mayo Clinic Vascular Disease Biorepository consisting of 1022 AAA cases and 7750 controls. Additional adjustments in the association were made to account for the presence of smoking and a family history of AAA. Once the best performing PRS_AAA_ was identified, we validated our results using AAA data from 3 additional population-based datasets: (1) prevalent AAA data from MVP release 3.0 using 1656 AAA cases and 44 908 controls of European ancestry and 718 AAA cases and 46 380 controls of African ancestry independent from the individuals in the MVP discovery GWAS and specifically designated for PRS analysis; (2) a set of 195 AAA cases and 9348 controls from the BioMe Biobank Program; and (3) a set of 396 AAA cases and 9835 controls from the Penn Medicine Biobank. We tested the association of the continuous PRS_AAA_ with prevalent AAA in each cohort and identified the prevalence of AAA in those individuals with the highest 5% of polygenic risk. We calculated the prevalence of AAA in each PRS quintile and in those individuals with the highest 5% of polygenic risk among men aged 50 or older, because individuals in their 6th decade of life or older are significantly more likely to have AAA.^25^ Last, we tested the association of our continuous PRS_AAA_ while including 6 traditional AAA risk factors in the association model: smoking, hypertension, low-density lipoprotein cholesterol with statin adjustment, high-density lipoprotein cholesterol, triglycerides, and coronary artery disease as a marker of atherosclerosis burden.

### Statistical Analysis

In our primary discovery analysis, genotyped and imputed DNA sequence variants in individuals of European ancestry were tested for association with AAA using logistic regression adjusting for age, sex, and 5 principal components of ancestry assuming an additive model using the SNPTESTv2.5.3 statistical software program.

For variants with suggestive AAA associations (*P*<10^−5^), we sought replication of our findings from either an in silico query from the 2016 AAA meta-analysis^7^ (stage 2a) or the HUNT/eMERGE Network datasets (stage 2b). In HUNT, the GWAS was performed using a logistic mixed model implemented in SAIGE (Scalable and Accurate Implementation of Generalized Mixed Model).^26^ Covariates in the model were sex, history of cardiovascular event as defined from HUNT questionnaires, history of hypertension, history of diabetes mellitus, myocardial infarction, angina pectoris, and smoking status (ever/never). In the eMERGE Network, association testing was performed using the PLATO software^27^ adjusted for year of birth, sex, 6 principal components, and sample collection site. Analyses were restricted to eMERGE Network locations with adult participants and those contributing cases to the analysis. Discovery and replication results were combined using an inverse variance–weighted fixed-effects method as implemented in the METAL software program.^28^ We did not attempt replication of any variant in either stage and required *P*<0.05 with concordant direction of effect for successful replication. We then combined statistical evidence across MVP and the replication studies and set a significance threshold of *P*<5×10^−8^ (genome-wide significance). Novel loci were defined as being >500 000 base pairs away from a known AAA genome-wide associated lead variant. Linkage disequilibrium information from the 1000 Genomes Project^29^ was used to determine independent variants when a locus extended beyond 500 000 base pairs. All logistic regression values of *P* were 2-sided.

In the PheWAS analysis, DNA sequence variants were tested using logistic regression adjusting for age, sex, and 5 principal components. Variants were declared to be significantly associated with the disease if they met *P*<1.7×10^−6^ (*P*<0.05/[1249 diseases × 24 variants]). In our genetic correlation analyses, traits were declared to be significant if they demonstrated a correlation *P*<0.005 (*P*<0.05/10 AAA risk factors).

In our MR analyses, a random-effects inverse variance–weighted method was used as the main analysis, with sensitivity analyses performed for the statistically significant associations as described previously. We set a 2-sided *P*<0.01 (0.05/5 traits) for statistical significance.

In addition, χ^2^ tests of independence were used to examine whether the occurrence of AAA with aneurysms in other vascular territories was more frequent than would be expected by chance. In the association analysis of AAA risk variants with aneurysms in other vascular territories, we performed logistic regression adjusting for age, sex, and 5 principal components of ancestry assuming an additive model using the R statistical software program^30^ (R version 3.3). In our sensitivity analysis, risk variants were tested using the same logistic model with individuals with AAA excluded. Given the known prior association with AAA, we used a nominally significant *P*<0.05 to declare significance. All *P* values were 2-sided.

In our PRS analysis, logistic regression models were used to estimate odds ratios (ORs) and 95% CIs for the associations of each of the continuous PRSs (1 SD unit), first adjusting only for age, sex, and 5 principal components. Family history and smoking status were then included in the model as appropriate. We then performed logistic regression adjusting for age, sex, 5 principal components, statins, and 6 clinical risk factors for AAA in the association model. We calculated prevalence of AAA for the 5% of individuals with the highest PRS_AAA_ relative to the rest of the population and generated CIs using R (version 3.3). All *P* values were 2-sided.

## Results

The MVP discovery analysis comprised 7642 patients with AAA and 172 172 controls of European ancestry. Their baseline characteristics are presented in Table 1. Participants with AAA were more likely to be older, male, and prescribed statin therapy; to have a history of smoking; and to have type 2 diabetes mellitus. Through genotype imputation, we obtained 18.6 million DNA sequence variants for analysis. After discovery, a total of 549 variants at 16 loci met a genome-wide significance threshold (Figures II and III in the Data Supplement). We replicated all 10 previously described genome-wide AAA loci with association *P*<10^−5^ (Table 2 and Table I in the Data Supplement). The *9p21* variant rs4007642 was the top association result (49.5% frequency for the T allele; OR, 1.21 [95% CI, 1.17–1.25]; *P*=6.9×10^−29^).

**Table 1.**
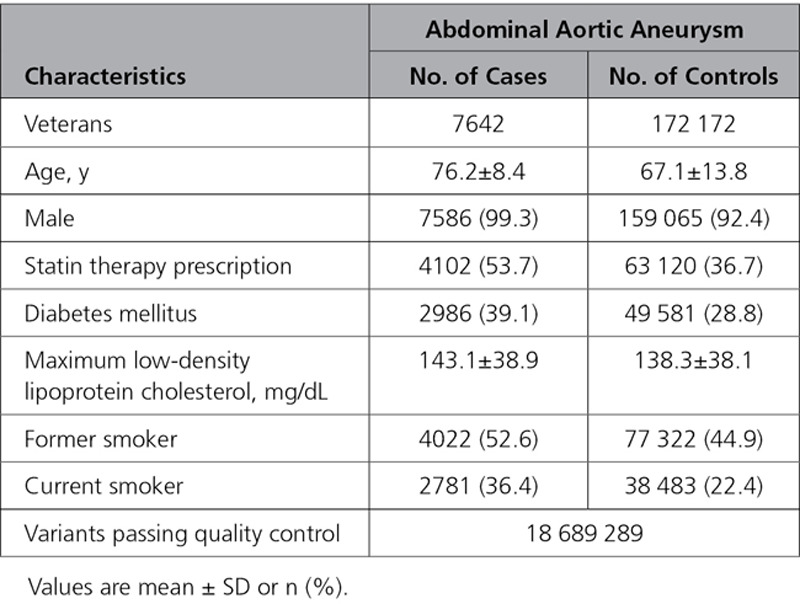
Demographic and Clinical Characteristics of Veterans in the Million Veteran Program Abdominal Aortic Aneurysm Genome-Wide Association Studies Analysis

**Table 2.**
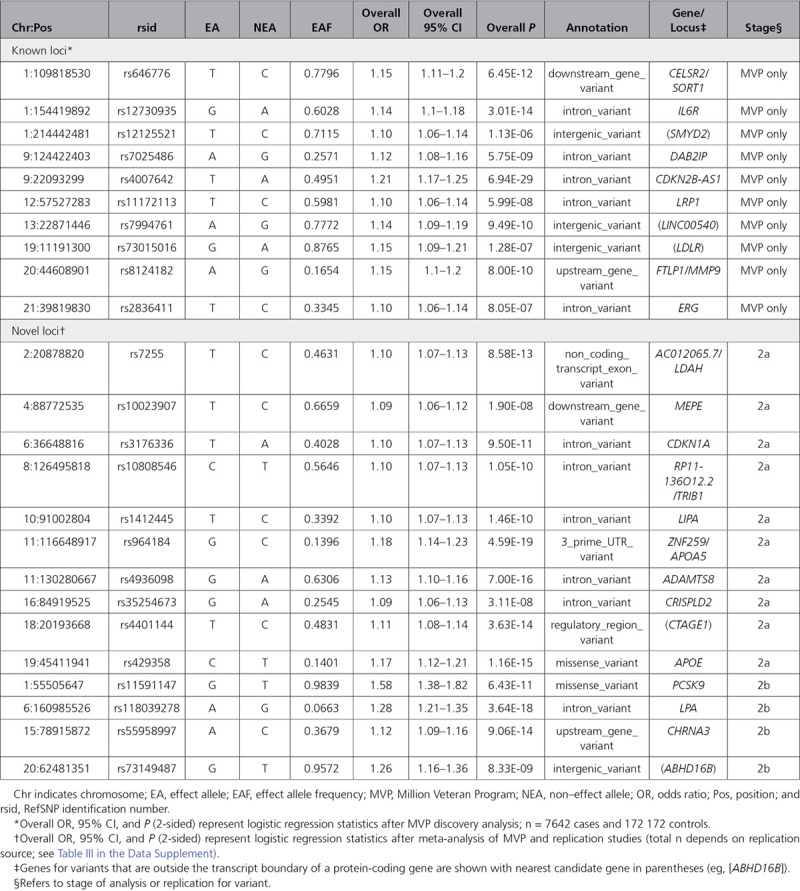
Risk Loci Associated With Abdominal Aortic Aneurysm in the MVP Genome-Wide Association Studies Discovery Analysis

A total of 70 lead variants at candidate novel loci demonstrated association *P*<10^−5^ in the MVP discovery analysis and were taken forward for replication. Of those, 28 were available for independent testing in the 2016 AAA meta-analysis, and the remaining were tested in the combined HUNT and eMERGE Network datasets. After independent replication, 14 novel loci exceeded genome-wide significance (*P*<5×10^−8^; Table 2 and Table II in the Data Supplement). The remaining variants either did not meet the prespecified *P*<0.05 with consistent direction of effect required for independent replication or did not surpass the genome-wide significance threshold after meta-analysis (Table III in the Data Supplement).

Understanding the full spectrum of phenotypic consequences of a given DNA sequence variant can help identify the mechanism by which a variant or gene leads to disease. Using a median of 65 distinct ICD-9 or ICD-10 EHR-derived diagnosis codes per participant, we tested each of the 24 AAA lead risk variants (14 newly identified in our study, 10 known) across 1249 disease phenotypes. In total, we identified 232 statistically significant (*P*<1.7×10^−6^) PheWAS associations across the 24 genetic variants. Thirteen of the loci replicated known associations with atherosclerosis in at least 1 arterial territory (peripheral, coronary, or cerebral). We also found that several of the DNA sequence variants associated with a range of known risk factors for AAA (Table IV in the Data Supplement). For example, we found 7 PheWAS associations with hyperlipidemia and hypertriglyceridemia. These loci have previously associated with either low-density lipoprotein cholesterol (*LDLR*, *PCSK9*, *SORT1*, *LPA*, *APOE*)^17^ or triglycerides (*APOA5*, *TRIB1*),^17^ both of which have been causally implicated in AAA development.^31^ The AAA risk allele for rs7255 near the lipid droplet-associated hydrogenase (*LDAH*) gene was associated with a decreased risk of hypercholesterolemia, suggesting that a mechanism other than increased serum cholesterol likely links the locus to aneurysmal degeneration. In *CHRNA3* (encoding cholinergic receptor nicotinic α-3), rs55958997 demonstrated associations with chronic obstructive pulmonary disease and ventilator dependence. This DNA sequence variant is strongly correlated (*R*^2^=0.83) with variants shown to predict nicotine dependence and a reduced likelihood for cigarette smoking cessation^32^ and appears to confer AAA risk entirely through its effect on smokers (Figure IV in the Data Supplement).

We next sought to more broadly understand the relationship of traditional AAA risk factors with disease at the genome-wide level. Analysis of shared heritability provides a mechanism to better understand the relationship of common variant risk across phenotypes.^16,33^ Using linkage disequilibrium score regression,^16^ we examined the genetic correlation between AAA and 10 known risk factors for disease and aneurysm rupture (Figure V in the Data Supplement). We found that 9 of the 10 risk factor traits were significantly correlated with AAA after Bonferroni correction (*P*<0.005), and all risk factors showed a consistent direction of effects between the epidemiologically posited association and the genetic correlation. Current smoking and coronary artery disease (a surrogate for atherosclerosis) demonstrated the strongest positive correlation (Table V in the Data Supplement). These results suggest a common genetic architecture for AAA and its individual risk factors.

**Figure 2.**
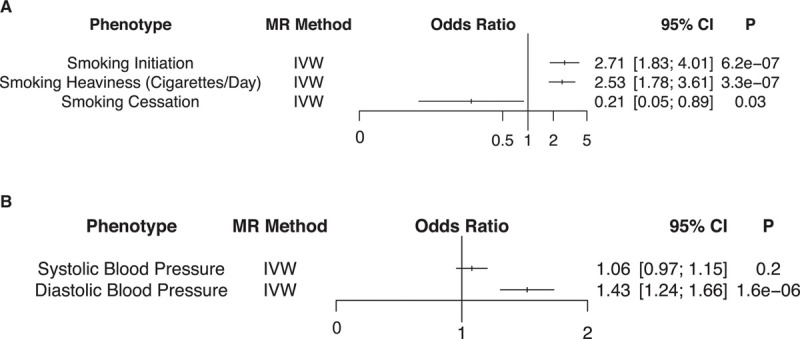
**Smoking and blood pressure mendelian randomization (MR) analyses.** Logistic regression association results of the (**A**) smoking and (**B**) blood pressure genetic instruments with the abdominal aortic aneurysm (AAA) outcome in 2-sample MR analyses. The smoking initiation and cessation odds ratios correspond to the change in AAA risk per 1 U log-odds increase in likelihood of the exposure, whereas the smoking heaviness odds ratio corresponds to the change in AAA risk per ≈5 cigarettes/d increase in smoking. The systolic and diastolic blood pressure odds ratios correspond to the change in AAA risk per 10-mm Hg increase in the blood pressure trait. Two-sided values of *P* are displayed. IVW indicates inverse variance–weighted.

In observational studies, lipids, smoking, and hypertension are modifiable, independent risk factors for AAA. We have previously examined the causal relationship between lipids and AAA,^31^ and here we performed MR analyses for 3 smoking phenotypes—smoking initiation, smoking heaviness, and smoking cessation—with AAA development. Genetic instruments of 84, 20, and 8 variants for the smoking initiation, smoking heaviness, and smoking cessation exposures, respectively, were derived from publicly available GSCAN data^20^ (Table VI in the Data Supplement). Using the inverse variance–weighted method, we observed that a genetically increased risk of ever being a regular smoker (OR 2.71 per 1 log-odds unit increase in risk of smoking initiation [95% CI, 1.83–4.01]; *P*=6.2×10^−7^) and a genetic increase in smoking heaviness (OR 2.53 per 1 U increase in daily smoking or ≈5 cigarettes/d [95% CI, 1.78–3.61]; *P*=3.3×10^−7^) were both associated with increased risk of AAA (Figure 2A). Our genetic instrument for likelihood of smoking cessation was found to be protective (OR 0.21 per 1 log-odds unit increase in likelihood of smoking cessation [95% CI, 0.05–0.89]; *P*=0.03), although this result did not survive Bonferroni correction. Our results remained robust to multiple sensitivity analyses, including the weighted median^22^ and leave-one-out analyses, as well as MR-PRESSO^24^ and MR-Egger^23^ tests for evidence of horizontal pleiotropy (Table VII and Figures VI and VII in the Data Supplement).

We then examined the effects of 2 blood pressure traits, SBP and DBP, with AAA through MR. We generated genetic instruments of 390 and 396 variants for the SBP and DBP traits, respectively, from the combined International Consortium for Blood Pressure and UK Biobank discovery GWAS analysis^21^ (Table VIII in the Data Supplement). We observed that a genetic 10 mm Hg increase in DBP was associated with an increased risk of AAA (OR, 1.43 per 10 mm Hg increase in DBP [95% CI, 1.24–1.66]; *P*=1.6×10^−6^; Figure 2B). In sensitivity analyses, the MR-PRESSO global test was significant for evidence of horizontal pleiotropy for DBP; however, the outlier-corrected results demonstrated nearly identical findings (Table IX and Figures VIII and IX in the Data Supplement). We did not detect a significant association between a genetic 10 mm Hg increase in SBP and AAA risk (OR, 1.06 per 10 mm Hg increase in SBP [95% CI, 0.97–1.15]; *P*=0.2).

**Figure 3.**
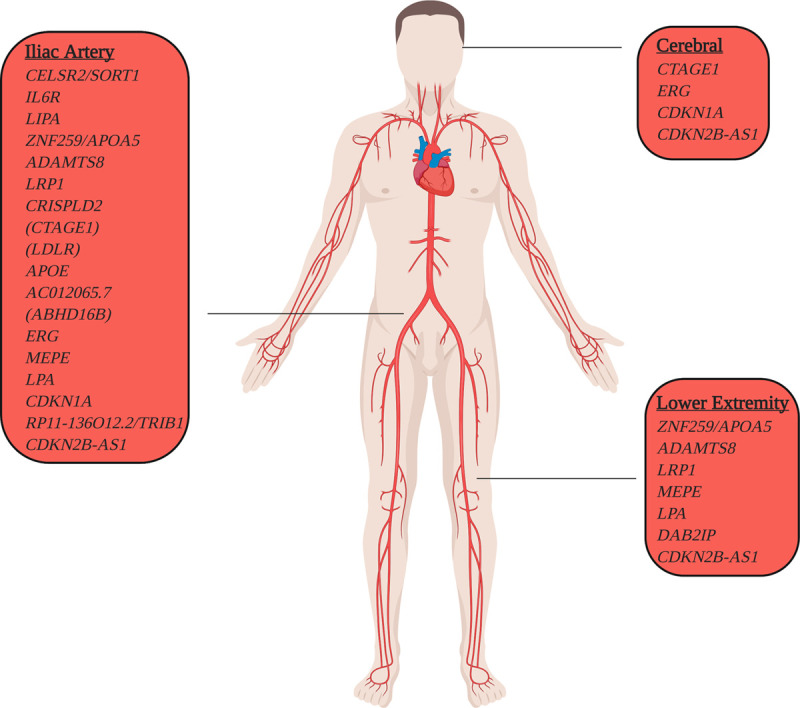
**Abdominal aortic aneurysm (AAA) risk loci with aneurysmal pleiotropy.** AAA risk loci with evidence of association with aneurysms in other vascular territories. Within our Million Veteran Program European cohort, we identified 659 patients with cerebral, 520 with lower extremity, and 844 with iliac aneurysm. We tested the 24 sentinel AAA risk variants for association with cerebral, lower extremity, and iliac aneurysms using logistic regression after adjusting for age, sex, and principal components of ancestry. AAA risk loci are displayed based on their pleiotropic aneurysmal association (*P*<0.05).

Next, we examined the strength of the relationship between AAA and aneurysms in other vascular territories. In total, we identified 659 cerebral, 520 lower extremity, and 844 iliac aneurysm cases among MVP participants of European ancestry. Whereas a significant relationship was observed for AAA and all 3 aneurysm types, we observed a stronger association among AAA and iliac aneurysms (χ^2^ *P*<1×10^−300^) as well as AAA and lower extremity aneurysms (χ^2^ *P*<1×10^−300^) than AAA and cerebral aneurysms (χ^2^ *P*=6×10^−12^). A total of 66% of patients with AAA were also noted to have an iliac aneurysm, 44% a lower extremity aneurysm, and 9.7% a cerebral aneurysm (Table X in the Data Supplement). We then extended our analysis to examine the underlying genetics of these diseases. For each of the 24 lead AAA risk variants, we tested their association with cerebral, iliac artery, and lower extremity aneurysms. We observed that 4 AAA risk variants demonstrated at least nominal association (*P*<0.05) with cerebral aneurysms, 7 with lower extremity aneurysms, and 18 with iliac aneurysms (Figure 3 and Table XI in the Data Supplement). In a sensitivity analysis, we examined the association of the AAA risk variants with isolated iliac, lower extremity, and cerebral aneurysms by excluding individuals with a diagnosis of AAA from the analysis. We noted a greater attenuation of association for the AAA risk variants with iliac artery and lower extremity aneurysms than for cerebral aneurysms, likely driven by the greater overlap of AAA diagnosis for aneurysms of the iliac and lower extremity vessels than in the cerebral territory (Table XII in the Data Supplement).

**Figure 4.**
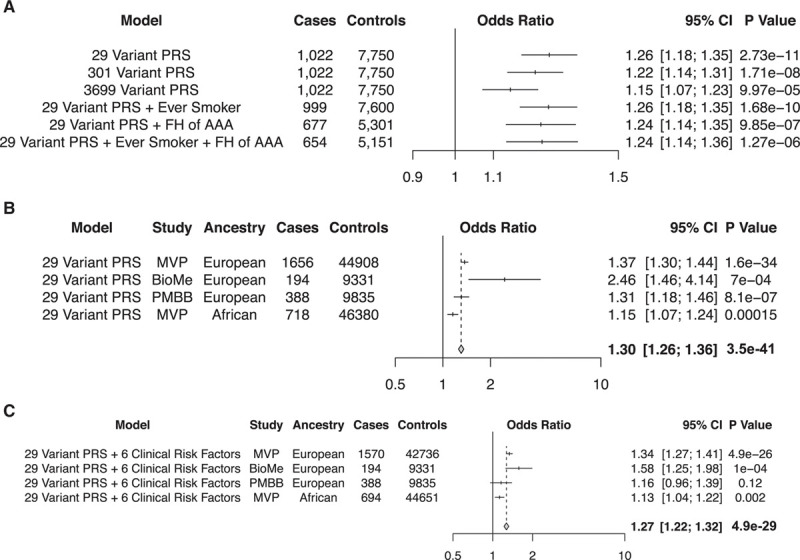
**Genome-wide polygenic risk score (PRS) for abdominal aortic aneurysm (AAA)**. **A**, AAA odds ratios and 2-sided *P* values in an ascertained case-control cohort from the Mayo Clinic Vascular Disease Biorepository per 1 SD increase in PRS_AAA_ for 3 separate PRSs. The addition of smoking or family history (FH) to the association model only slightly attenuated the effect estimate observed. Case/control counts in the models including smoking and FH are reduced after accounting for missingness for these variables across the cohort. **B**, AAA odds ratios and 2-sided *P* values in 4 different populations (Europeans in the Million Veteran Program [MVP], BioMe, and Penn Medicine Biobank [PMBB]; Africans in the MVP) per 1 SD increase in PRS_AAA_. Results were combined in an inverse variance–weighted fixed effects meta-analysis. **C**, AAA odds ratios and 2-sided *P* values in 4 different populations (Europeans in the MVP, BioMe, and PMBB; Africans in the MVP) per 1 SD increase in PRS_AAA_, accounting for 6 additional clinical risk factors in the association model. Results were combined in an inverse variance–weighted fixed effects meta-analysis.

We aimed to examine the contribution of polygenic inheritance on AAA risk. We hypothesized that those at the right tail of the normally distributed AAA PRS (highest 5%) would identify a population at increased risk of AAA who may be candidates for screening. We generated 3 distinct AAA PRSs using a pruning and thresholding method from European MVP release 2.1 AAA summary statistics (*R*^2^<0.0001 for each score, association *P*<0.01, *P*<1×10^−4^, or *P*<1×10^−6^; Table XIII in the Data Supplement). We first tested the associated AAA risk for each of the PRS_AAA_ using ascertained AAA case–control cohort data from the Mayo Clinic Vascular Disease Biorepository^34^ consisting of 1022 AAA cases and 7750 controls. Demographic and clinical characteristics of this European ancestry cohort are depicted in Table XIV in the Data Supplement. We observed that the 29 variant score (*R*^2^<0.0001, *P*<1×10^−6^; score 1) performed best based on its effect estimate and *P* value of association, with a 1-SD increase in PRS_AAA_ associated with a 26% increased risk of AAA (OR_PRS_, 1.26 [95% CI, 1.18–1.36]; *P*_PRS_=2.7×10^−11^; Figure 4A). Including family history of AAA or smoking in the model only slightly attenuated the association effect estimate for AAA (OR_PRS+FH+__smoking_, 1.24 [95% CI, 1.14–1.35]; *P*_PRS_=1.27×10^−6^; Figure 4A).

We sought validation of our PRS findings using AAA data from 3 additional population-based datasets: (1) prevalent AAA data from MVP release 3.0, which comprised 1656 AAA cases and 44 908 controls of European ancestry and 718 AAA cases and 46 380 controls of African ancestry that were entirely independent from the individuals in the MVP discovery GWAS; (2) a set of 195 AAA cases and 9348 controls from the BioMe Biobank Program; and (3) a set of 396 AAA cases and 9835 controls from the Penn Medicine Biobank (Table XV in the Data Supplement). We observed that the 29-variant PRS_AAA_ was associated with AAA in all of the cohorts, though the effect estimate was diminished most in individuals of African ancestry (Figure 4B). In addition, among European individuals with high polygenic risk in each cohort (highest 5%), we noted an AAA prevalence of 5.9% (95% CI, 5.1–6.7%) for all individuals and 8.6% (95% CI, 7.3–9.8%) among men aged 50 or older (Table XVI in the Data Supplement). The prevalence estimates observed in individuals of European ancestry were greater than those found in individuals of African ancestry (1.7% among all individuals in the PRS top 5% and 2.5% among men aged 50 or older). Prevalence results stratified by PRS quintile are depicted in Table XVII in the Data Supplement.

We then included 6 clinical AAA risk factors in the association model to assess whether the PRS represented an orthogonal risk factor beyond what could be used to predict AAA risk on clinical grounds alone. We observed an attenuation in both effect estimate and association *P* value but the results remained statistically significant (Figure 4C).

## Discussion

We identified 14 novel loci and examined the overlap of AAA genetic variation with its risk factors at the genome-wide level. We tested AAA lead risk variants with risk of aneurysms in other vascular territories, and through MR, we demonstrate that DBP, as opposed to SBP, likely has a causal relationship with AAA development. Last, we developed a genome-wide PRS for AAA that identifies a subset of the population at greater risk for AAA independent of known smoking and family history risk factors.

These findings permit several conclusions. First, our findings lend human genetic support to DBP, as opposed to SBP, being of greater significance in the pathogenesis of AAA. Observational epidemiologic data from the CALIBER study (Cardiovascular Research Using Linked Bespoke Studies and Electronic Health Records) demonstrated that increased DBP was associated with incident AAA^35^ and that unlike other atherosclerotic syndromes, elevated SBP was not predictive of AAA risk. Our MR results support these findings and suggest a causal role for elevated DBP in aneurysm development. Given that diastole represents ≈2/3 of the cardiac cycle, our results suggest that abdominal aortic exposure to a hypertensive environment over a long period of time may be more important than exposure to brief, elevated peaks in blood pressure in promoting aneurysmal degeneration. Whereas many of the same causal pathways for atherosclerosis have been implicated in AAA, including dyslipidemia and tobacco exposure, a key difference appears to be that genetically increased SBP—previously shown to predispose to an increased risk for atherosclerosis^36^—is not as important in the pathogenesis of AAA.

Second, our results highlight biological similarities and differences among aneurysms of the aortic, cerebral, lower extremity, and iliac systems. Previous analyses demonstrated that cerebral aneurysms may be observed in up to 10% of patients with AAA,^37^ and estimates for lower extremity and iliac aneurysms among those with AAA approach 14%^38^ and 40%,^39^ respectively. In our population-based genetic analysis, we observed a much greater amount of disease overlap between AAA and iliac or lower extremity aneurysms than previously reported, with the shared genetic risk associations reflecting this distribution. Our findings support the concept that the biology underlying AAA, iliac, and lower extremity aneurysms are likely closely related, whereas cerebral aneurysms appear to be a physiologic entity divergent in genetic origin. Whereas the final common pathway of arterial wall degeneration is common to all 4 diseases, these results suggest that therapies targeting AAA are more likely to be efficacious for preventing aneurysmal disease in the lower extremity and iliac vascular beds.

Last, our data identify a role for polygenic risk prediction in the targeting of asymptomatic individuals for AAA screening. AAA remains underdiagnosed in the general population. A 2013 report examining Medicare data demonstrated that up to 40% of individuals with AAA were diagnosed late in their disease course, and late diagnosis was associated with an increased likelihood of rupture at the time of repair.^40^ The US Preventative Service Task Force recently released updated guidelines^41^ regarding the utility of screening asymptomatic patients on the basis of pooled data from 4 randomized control trials.^42–45^ Across these studies, a net reduction in aneurysm-related mortality and incidence of rupture was observed by screening men >65 years of age, leading to the recommendation to screen individuals aged 65 to 75 with a history of smoking with a single visceral ultrasound. This recommendation was based on the assumption that 6% to 7% of individuals in this cohort will have an identified AAA.^25^ Here, through the use of a 29-variant PRS_AAA_, we identified a subset of the European ancestry population who had high genetic risk and an AAA prevalence similar to (5.9% across the top 5% PRS_AAA_) or greater than (8.6% among men over 50 in the top 5% PRS_AAA_) that observed in screening trials informing current guidelines. Furthermore, a family history of AAA, often touted as an equivalent means of assessing genetic risk, only slightly attenuated the PRS association. Results from our study suggest that PRSs represent an orthogonal source of disease risk not captured by standard AAA risk factors, and testing individuals with a high PRS_AAA_ through a noninvasive abdominal duplex study may increase the yield of AAA screening, particularly once genotyping becomes standard of care in healthcare systems and can be performed at nominal cost.

These results also underscore an important issue with respect to applicability of polygenic scoring across ancestry groups. To date, the overwhelming majority of PRSs have been developed using summary statistics from patients with European ancestry. Whereas the 29-variant score constructed here associated with AAA in individuals with African ancestry, the attenuated effect estimate could limit its predictive accuracy. As the field of genetics moves forward, it is critical that large-scale association studies include individuals of diverse genetic backgrounds to limit the potential for ethnic disparities in precision medicine.

Our study should be interpreted within the context of its limitations. First, our AAA phenotype is based on EHR data and may result in misclassification of case status. Such misclassification should, however, reduce statistical power for discovery and on average bias results toward the null. Second, although those with the highest PRS_AAA_ are at increased risk for AAA, the PRS mechanism of action represents a combination of many causal risk factors, rather than a single pathway that leads to disease. However, assessment of individual risk can aid in identifying those at highest risk for AAA and more likely to obtain benefit from screening, regardless of mechanism. Third, the Veterans Health Administration healthcare system contains a much higher proportion of men than the general population, and our ability to detect female-specific associations was more limited. However, AAA is a disease process that overwhelmingly affects men,^25^ and our results should therefore still be generalizable to patients with AAA.

We identified 14 novel genomic loci associated with AAA risk, explored the phenotypic consequences of blood pressure on AAA, identified AAA risk variants associated with aneurysms in other vascular beds, and generated a PRS for AAA that may improve the current screening paradigm once genotyping costs are further reduced in the future. Our data provide mechanistic insights into the genetic architecture of AAA that can inform clinical care.

## Sources of Funding

This work was supported by funding from the Department of Veterans Affairs Office of Research and Development, Million Veteran Program Grant MVP000; Department of Veterans Affairs awards I01-01BX03340 (Drs Cho and Wilson), I01-BX003362 (Drs Chang and Tsao), and IK2-CX001780 (Dr Damrauer); Veterans Affairs Informatics and Computing Infrastructure (VINCI) grant VA HSR RES 130457 (Dr DuVall); and National Institutes of Health grant K08HL140203 (Dr Natarajan). Phase III of the eMERGE Network (electronic Medical Records and Genomics) was initiated and funded by the National Human Genome Research Institute through the following grants: U01HG8657 (Kaiser Washington/University of Washington), U01HG8685 (Brigham and Women’s Hospital), U01HG8672 (Vanderbilt University Medical Center), U01HG8666 (Cincinnati Children’s Hospital Medical Center), U01HG6379 (Mayo Clinic), U01HG8679 (Geisinger Clinic), U01HG8680 (Columbia University Health Sciences), U01HG8684 (Children’s Hospital of Philadelphia), U01HG8673 (Northwestern University), U01HG8701 (Vanderbilt University Medical Center serving as the Coordinating Center), U01HG8676 (Partners Healthcare/Broad Institute), and U01HG8664 (Baylor College of Medicine). Phase I and II of the eMERGE Network was initiated and funded by the National Human Genome Research Institute through the following grants: U01HG006828 (Cincinnati Children’s Hospital Medical Center/Boston Children’s Hospital), U01HG006830 (Children’s Hospital of Philadelphia), U01HG006389 (Essentia Institute of Rural Health, Marshfield Clinic Research Foundation, and Pennsylvania State University), U01HG006382 (Geisinger Clinic), U01HG006375 (Group Health Cooperative/University of Washington), U01HG006379 (Mayo Clinic), U01HG006380 (Icahn School of Medicine at Mount Sinai), U01HG006388 (Northwestern University), U01HG006378 (Vanderbilt University Medical Center), U01HG006385 (Vanderbilt University Medical Center, serving as the Coordinating Center), U01HG004438 (Center for Inherited Disease Research, serving as a Genotyping Center), and U01HG004424 (the Broad Institute, serving as a Genotyping Center).

## Disclosures

Dr Klarin has received consulting fees from Regeneron Pharmaceuticals unrelated to the work in this article. Dr DuVall reports grants and nonfinancial support from the Department of Veterans Affairs during the conduct of the study; and grants from AbbVie Inc, Amgen Inc, Anolinx LLC, Astellas Pharma Inc, AstraZeneca Pharmaceuticals LP, Boehringer Ingelheim International GmbH, Celgene Corporation, Eli Lilly and Company, Genentech Inc, Genomic Health Inc, Gilead Sciences Inc, GlaxoSmithKline PLC, Innocrin Pharmaceuticals Inc, Janssen Pharmaceuticals Inc, Kantar Health, Myriad Genetics Laboratories Inc, Novartis International AG, and Parexel International Corporation outside the submitted work. Dr Damrauer receives research support to his institution from RenalytixAI and is a paid consultant for Calico Labs. Dr Kathiresan is a founder of Maze Therapeutics, Verve Therapeutics, and San Therapeutics; holds equity in Catabasis and San Therapeutics; is a member of the scientific advisory boards for Regeneron Genetics Center and Corvidia Therapeutics; served as a consultant for Acceleron, Eli Lilly, Novartis, Merck, NovoNordisk, Novo Ventures, Ionis, Alnylam, Aegerion, Huag Partners, Noble Insights, Leerink Partners, Bayer Healthcare, Illumina, Color Genomics, MedGenome, Quest, and Medscape; and reports patents related to a method of identifying and treating a person having a predisposition to or afflicted with cardiometabolic disease (20180010185) and a genetics risk predictor (20190017119).

## Supplemental Materials

Supplemental Methods

Data Supplement Figures I–IX

Data Supplement Tables I–XVII

Data Supplement Excel File I

References 46–54

## Supplementary Material


