# Supplementary material for: Genetic Architecture of Abdominal Aortic Aneurysm in the Million Veteran Program
Source: Circulation. 2020 Sep 28;142(17):1633–46. doi: 10.1161/CIRCULATIONAHA.120.047544 (PMC7580856; doi:10.1161/CIRCULATIONAHA.120.047544)
Supplement: Supplementary file 1 [file cir-142-1633-s001.pdf]

**SUPPLEMENTAL MATERIAL**

# Genetic Architecture of Abdominal Aortic Aneurysm in the Million Veteran Program

Derek Klarin et al.

**Short Title: GWAS of AAA in MVP**

**Corresponding Author**
Philip S. Tsao, Ph.D.
VA Palo Alto Health Care System, Palo Alto, California, USA
Department of Medicine, Stanford University School of Medicine, Stanford, California, USA
Email: philip.tsa@va.gov or ptsao@stanford.edu
Tel:
(650) 493-5000 x62991
(650) 725-2178 (fax)

## Expanded Methods

*Genetic Data and Quality Control*
DNA extracted from whole blood was genotyped in the Million Veteran Program (MVP) using a customized Affymetrix Axiom biobank array, the MVP 1.0 Genotyping Array[46]. Veterans (U.S. military personnel) of two mutually exclusive ethnic groups were identified for analysis: 1) non-Hispanic whites (European ancestry) for discovery and PRS analysis, and 2) non-Hispanic blacks (African ancestry) for PRS analysis. Prior to imputation, variants that were poorly called or that deviated from their expected allele frequency based on reference data from the 1000 Genomes Project[29] were excluded. After pre-phasing using EAGLE v2[47], genotypes from the 1000 Genomes Project[29] phase 3, version 5 reference panel were imputed into MVP participants via Minimac3 software[48]. Ethnicity-specific principal component analysis was performed using the EIGENSOFT v6 software[49]. Participants were then divided into two mutually exclusive ethnic groups based on self-identified race/ethnicity and admixture analysis using the ADMIXTURE v1.3 software[50]: 1) non-Hispanic whites (self-identified as "non-Hispanic," "white," and > 80% genetic European ancestry) and 2) non-Hispanic blacks (self-identified as "non-Hispanic," "black," and > 50% genetic African ancestry).

In MVP, sample and variant quality control was performed as previously described[31]. In brief, duplicate samples, those with more heterozygosity than expected, an excess (>2.5%) of missing genotype calls, or discordance between genetically inferred sex and phenotypic gender were excluded. In addition, one individual from each pair of related individuals (kinship > 0.0884 as measured by the KING 2.0 software[51]) were removed. In total, we identified 227,817 European participants for AAA discovery and PheWAS analysis from MVP release 2.1. For our PRS analysis, an additional, independent tranche of 1,656 AAA cases/44,908 controls of European ancestry and 718 AAA cases/46,380 controls of African ancestry were used after the above quality control metrics were applied.

Following imputation, variant level quality control was performed using the EasyQC R package (www.R-project.org), and exclusion metrics included: ancestry specific Hardy-Weinberg equilibrium $P < 1x10^{-20}$, posterior call probability < 0.9, imputation quality < 0.3, minor allele frequency (MAF) < 0.0003, call rate < 97.5% for common variants (MAF > 1%), and call rate < 99% for rare variants (MAF < 1%). Variants were also excluded if they deviated > 10% from their expected allele frequency based on reference data from the 1000 Genomes Project. Following variant level quality control, we obtained 18.6 million DNA sequence variants for analysis.

We sought replication from either the 2016 AAA meta-analysis[7] or the combined HUNT/eMERGE datasets. In the 2016 AAA meta-analysis, lead DNA sequence variants were replicated *in silico* from the summary statistics generated at the time of analysis. Data were included only if the variant was available within each study in the overall meta-analysis. In HUNT, DNA from 71,860 samples was genotyped using one of three different Illumina HumanCoreExome arrays (HumanCoreExome12 v1.0, HumanCoreExome12 v1.1 and UM HUNT Biobank v1.0). We excluded samples that failed to reach a 99% call rate, had contamination > 2.5% as estimated with BAF Regress, large chromosomal copy number variants, lower call rate of a technical duplicate pair and twins, gonosomal constellations other than XX and XY, or whose

inferred sex contradicted the reported gender. Samples that passed quality control were analyzed in a second round of genotype calling following the Genome Studio quality control protocol. Genomic position, strand orientation and the reference allele of genotyped variants were determined by aligning their probe sequences against the human genome (build 37) using BLAT. PLINK v1.90[52] was then used to exclude variants if their probe sequences could not be perfectly mapped, cluster separation was < 0.3, Gentrain score < 0.15, showed deviations from Hardy Weinberg equilibrium in unrelated samples of European ancestry with p-value < 0.0001, had a call rate < 99%, or another assay with higher call rate genotyped the same variant. Ancestry of all samples was inferred by projecting all genotyped samples into the space of the principal components from 938 unrelated individuals of the Human Genome Diversity Project reference panel. The different arrays were harmonized by reducing to a set of overlapping variants and excluding variants that showed frequency differences > 15% between data sets, or that were monomorphic in one and had MAF > 1% in another data set. The resulting genotype data were phased using EAGLE v2[47] imputation was performed on samples of recent European ancestry using Minimac[48] and a merged reference panel that was constructed by combining the Haplotype Reference Consortium (HRC)[53] panel and a local reference panel based on 2,202 whole-genome sequenced HUNT study participants. We excluded variants with rsq < 0.3 resulting in over 24.9 million well-imputed variants for single variant association analysis. In eMERGE, genotyping, quality control, and HRC imputation have been previously described[54].

*Diagnosis Codes and Definitions for AAA Phenotype Definition*

AAA cases were defined as the presence of two instances of any of the following ICD-9/10 codes in a participant's EHR:
ICD-9 441.3 - Abdominal aortic aneurysm, ruptured
ICD-9 441.4 - Abdominal aortic aneurysm, without rupture
ICD-10 I71.3 - Abdominal aortic aneurysm, ruptured
ICD-10 I71.4 - Abdominal aortic aneurysm, without rupture

Controls were defined as possessing zero occurrences of the aforementioned ICD codes, as well as zero occurrences of:

ICD-9 440 - Atherosclerosis
ICD-9 441 - Aortic aneurysm and dissection
ICD-9 442 - Other aneurysm
ICD-9 443 - Other peripheral vascular disease
ICD-9 444 - Arterial embolism and thrombosis
ICD-9 445 - Atheroembolism
ICD-9 446 - Polyarteritis nodosa and allied conditions
ICD-9 447 - Other disorders of arteries and arterioles
ICD-9 448 - Diseases of capillaries

ICD-10 I71 - Aortic aneurysm and dissection
ICD-10 I72 - Other aneurysm
ICD-10 I73 - Other peripheral vascular disease
ICD-10 I74 - Arterial embolism and thrombosis
ICD-10 I75 - Atheroembolism
ICD-10 I77 - Other disorders of arteries and arterioles
ICD-10 I78 - Diseases of capillaries
ICD-10 I79 - Disorders of arteries, arterioles and capillaries in diseases classified elsewhere

ICD-10 K55 - Vascular disorders of intestine

**Supplementary Figure I -** Overall Study Design

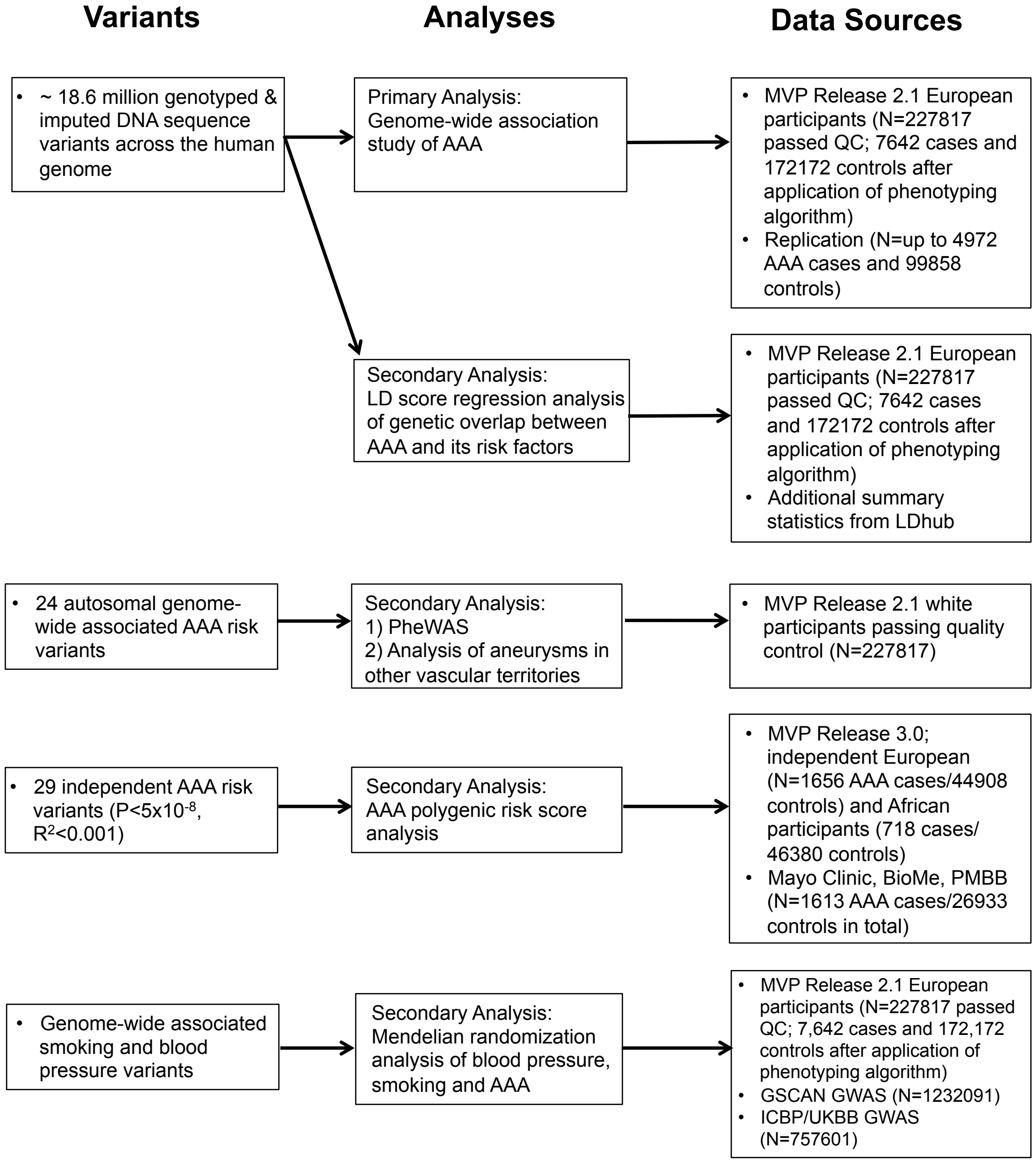


Supplementary Figure I - The primary analysis consisted of a genome-wide association study to identify novel AAA risk variants. Secondary analyses included: an analysis of AAA and overlap with its risk factors, a closer examination of AAA risk variants through PheWAS and their association with aneurysms in other vascular territories, generation and analysis of AAA polygenic risk scores, and Mendelian randomization analyses of smoking, blood pressure, and AAA.
Abbreviations: AAA, Abdominal Aortic Aneurysm; MVP, Million Veteran Program; PheWAS, Phenome-wide Association Study; QC, Quality Control; LD, Linkage Disequilibrium; PMBB, Penn Medicine Biobank; GSCAN, GWAS & Sequencing Consortium of Alcohol and Nicotine use; ICBP, International Consortium of Blood Pressure; UKBB, UK Biobank; GWAS, Genome-wide Association Study

**Supplementary Figure II** - Quantile-quantile plot for the discovery AAA GWAS in MVP

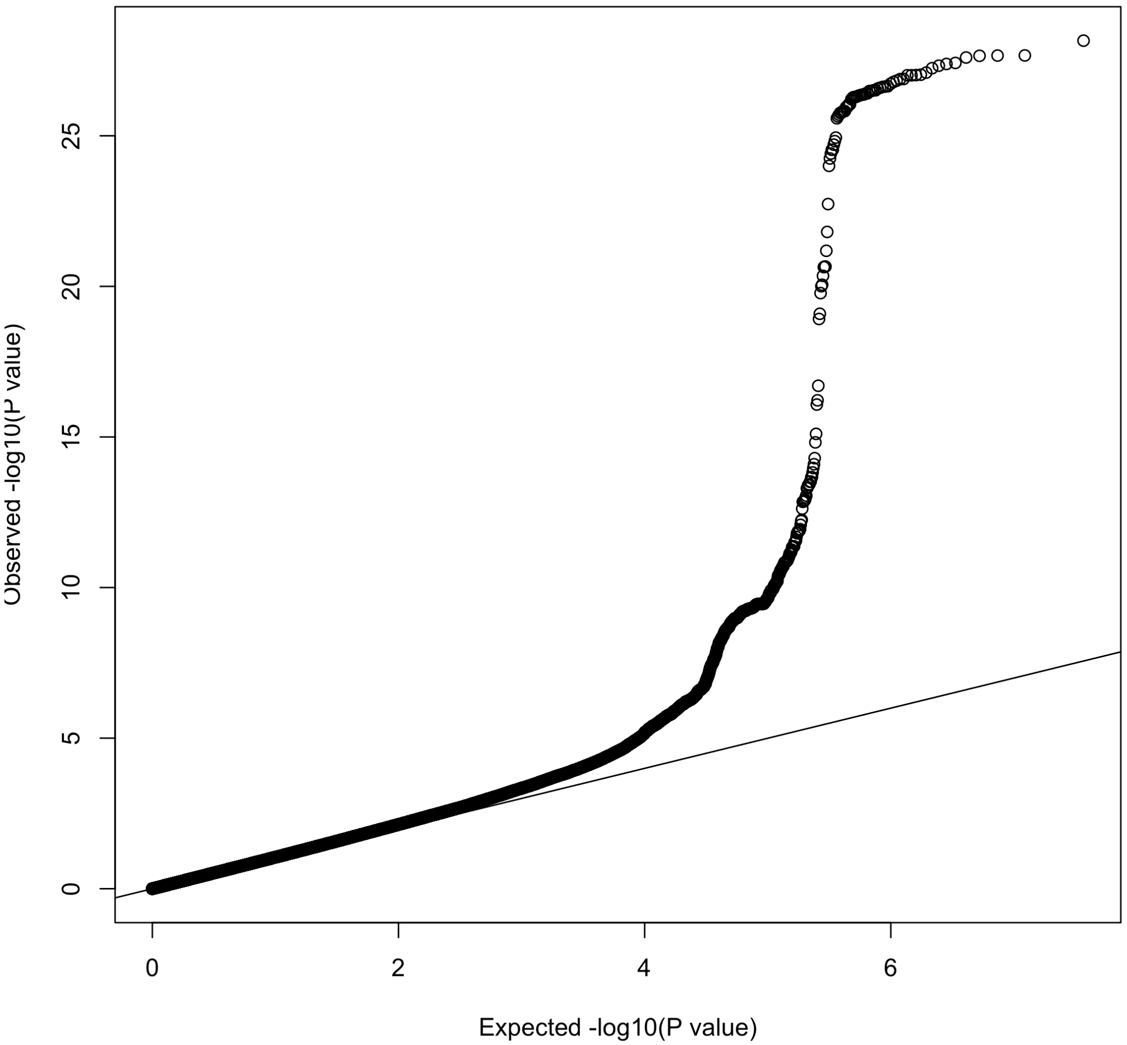


Supplementary Figure II - The expected logistic regression association P values versus the observed distribution of P values for AAA association are displayed. No systemic inflation was observed ($\lambda_{GC}$ = 1.07). All P values were two-sided. Abbreviations: AAA Abdominal Aortic Aneurysm; GWAS, Genome-wide Association Study; MVP, Million Veteran Program

**Supplementary Figure III -** Manhattan plot for the AAA GWAS

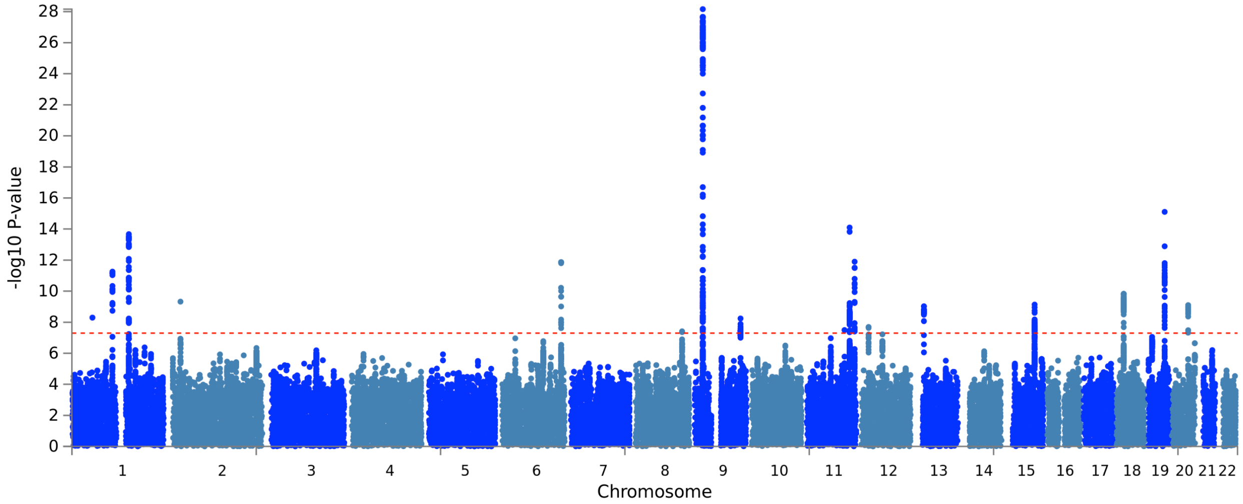


Supplementary Figure III - Plot of -log10(*P*) for association of genotyped and imputed variants by chromosomal position for all autosomal polymorphisms analyzed in the AAA GWAS. Logistic regression two-sided P values are displayed.
Abbreviations: AAA, Abdominal Aortic Aneurysm; GWAS, genome-wide association study

**Supplementary Figure IV -** Forest plot for association of the *CHRNA3* locus and AAA risk stratified by smoking status

| Gene | Disease | Smoking Status | Cases | Controls | Odds Ratio | 95% CI | P Value |
|---|---|---|---|---|---|---|---|
| CHRNA3 | AAA | Ever | 6,803 | 115,805 | 1.13 | [1.09; 1.17] | 5.7e−11 |
| CHRNA3 | AAA | Never | 776 | 47,085 | 1.02 | [0.92; 1.13] | 0.74 |

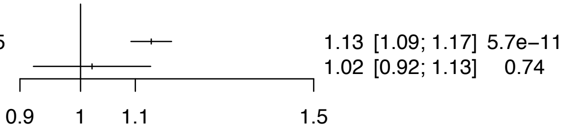


Supplementary Figure IV - When stratifying European MVP participants by smoking status (ever smokers vs. never smokers), nearly all the DNA sequence variant-AAA association signal resides within the ever smoker group. Previous reports of variation at the *CHRNA3* locus demonstrated that carriers of the AAA risk allele have a reduced likelihood of cigarette smoking cessation[55], and suggests that the AAA-*CHRNA3* association is driven by a greater burden of tobacco exposure in those who carry the nicotine dependence/AAA risk allele. Individuals without a smoking designation in the VHA electronic health record were excluded from this analysis. Logistic regression two-sided values of P are displayed.
Abbreviations: MVP, Million Veteran Program; AAA, Abdominal Aortic Aneurysm; VA, Veterans Affairs; CI, Confidence Interval

**Supplementary Figure V -** Genetic correlation of AAA with risk factors for disease and rupture

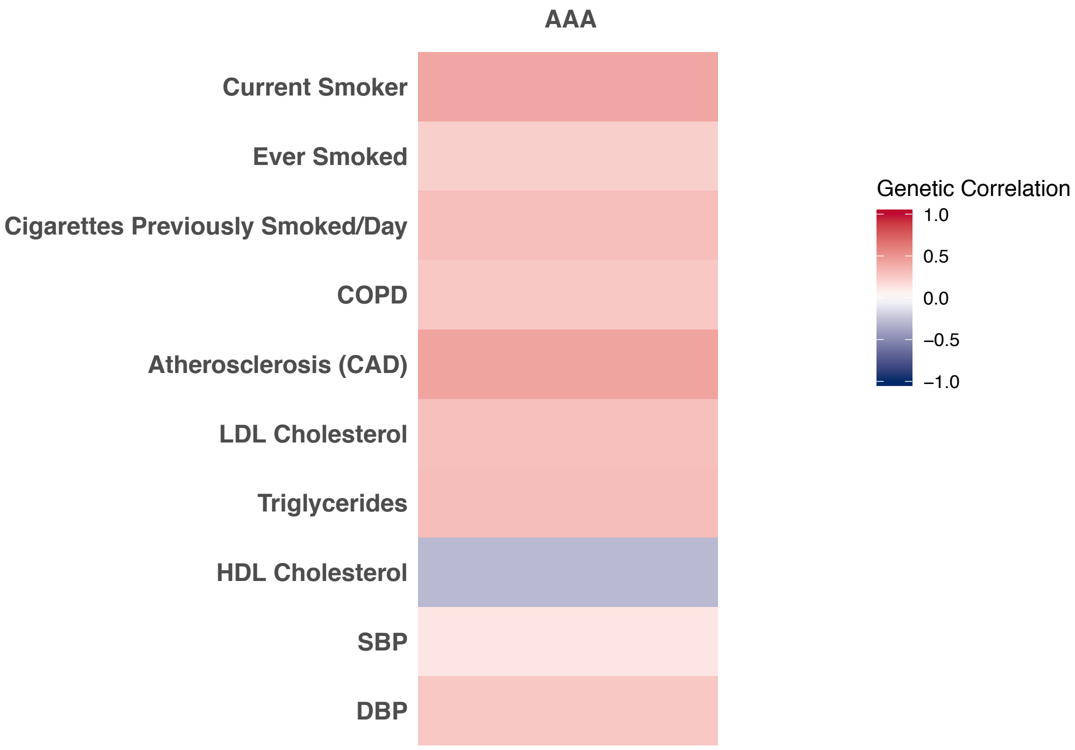


Supplementary Figure V - Using linkage disequilibrium score regression[16], summary statistics for AAA from the current study (N = 7,642 AAA cases and 172,172 controls) and 10 AAA risk factors were used to calculate a genetic correlation between traits ($r_g$). Nine of ten risk factors demonstrated a significant genetic correlation with AAA after Bonferroni correlation ($r_g$ $P < 0.005$ for all except systolic blood pressure). The genetic correlation values, associated standard errors, (two-sided) values of P, and sample sizes for each trait are depicted in Table V in the supplement.
Abbreviations: AAA, Abdominal Aortic Aneurysm; COPD, Chronic Obstructive Pulmonary Disease; CAD, Coronary Artery Disease; LDL, Low-Density Lipoprotein; HDL, High-Density Lipoprotein; SBP, Systolic Blood Pressure; DBP, Diastolic Blood Pressure

**Supplementary Figure VI -** Diagnostic leave-one-out plot for smoking exposure-AAA Mendelian randomization analysis

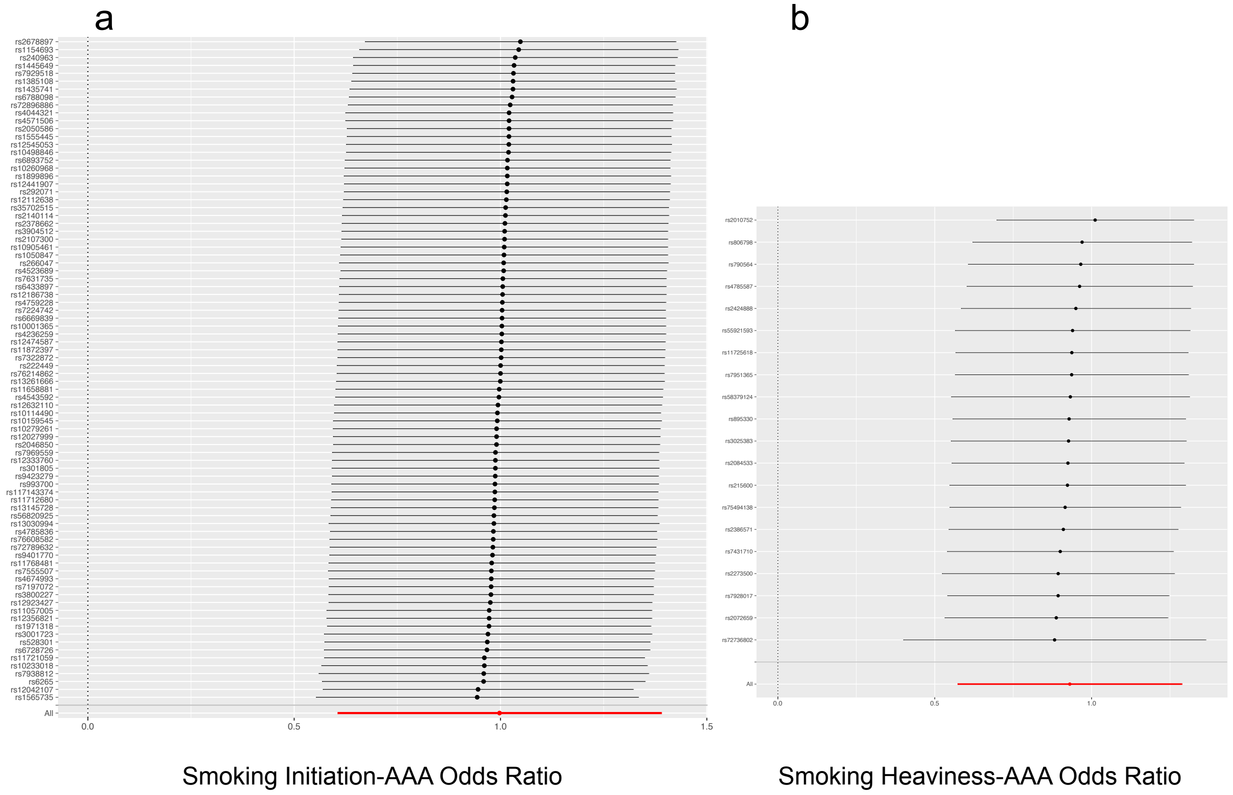


Supplementary Figure VI - Diagnostic leave-one-out plots were generated for each of the smoking exposures with a positive Mendelian randomization result - smoking initiation (a) and smoking heaviness (b) - with the AAA outcome. In each case, minimal evidence of a single association driving the inverse-variance weighted result was observed.

**Supplementary Figure VII -** Diagnostic funnel plot for smoking exposure-AAA Mendelian randomization analysis

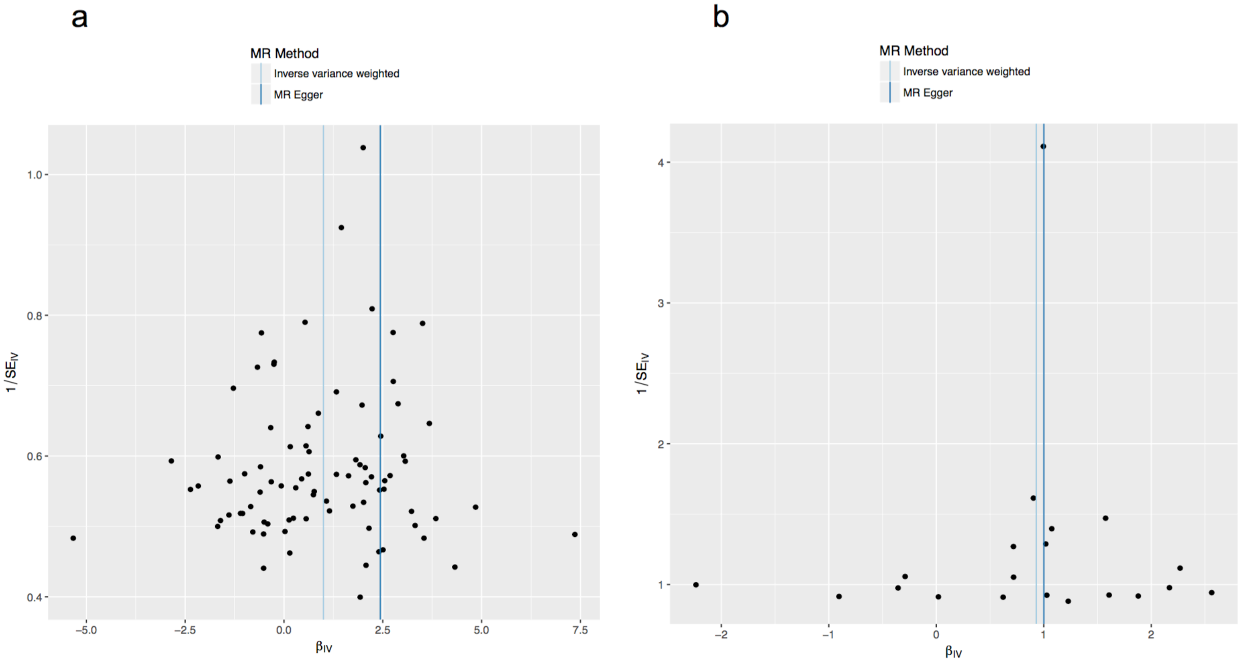


Supplementary Figure VII - A funnel plot depicting the reciprocal of the standard error (Y-axis) of the instrumental variable estimate against the instrumental variable estimates (X-axis) for smoking initiation (a) and smoking heaviness (b). Ideally, these plots should demonstrate a symmetric funnel, in which more precise estimates are less variable. Minimal asymmetry was observed
Abbreviations: MR, Mendelian Randomization; IV, Instrumental Variable

**Supplementary Figure VIII -** Diagnostic leave-one-out plot for diastolic blood pressure exposure-AAA Mendelian randomization analysis

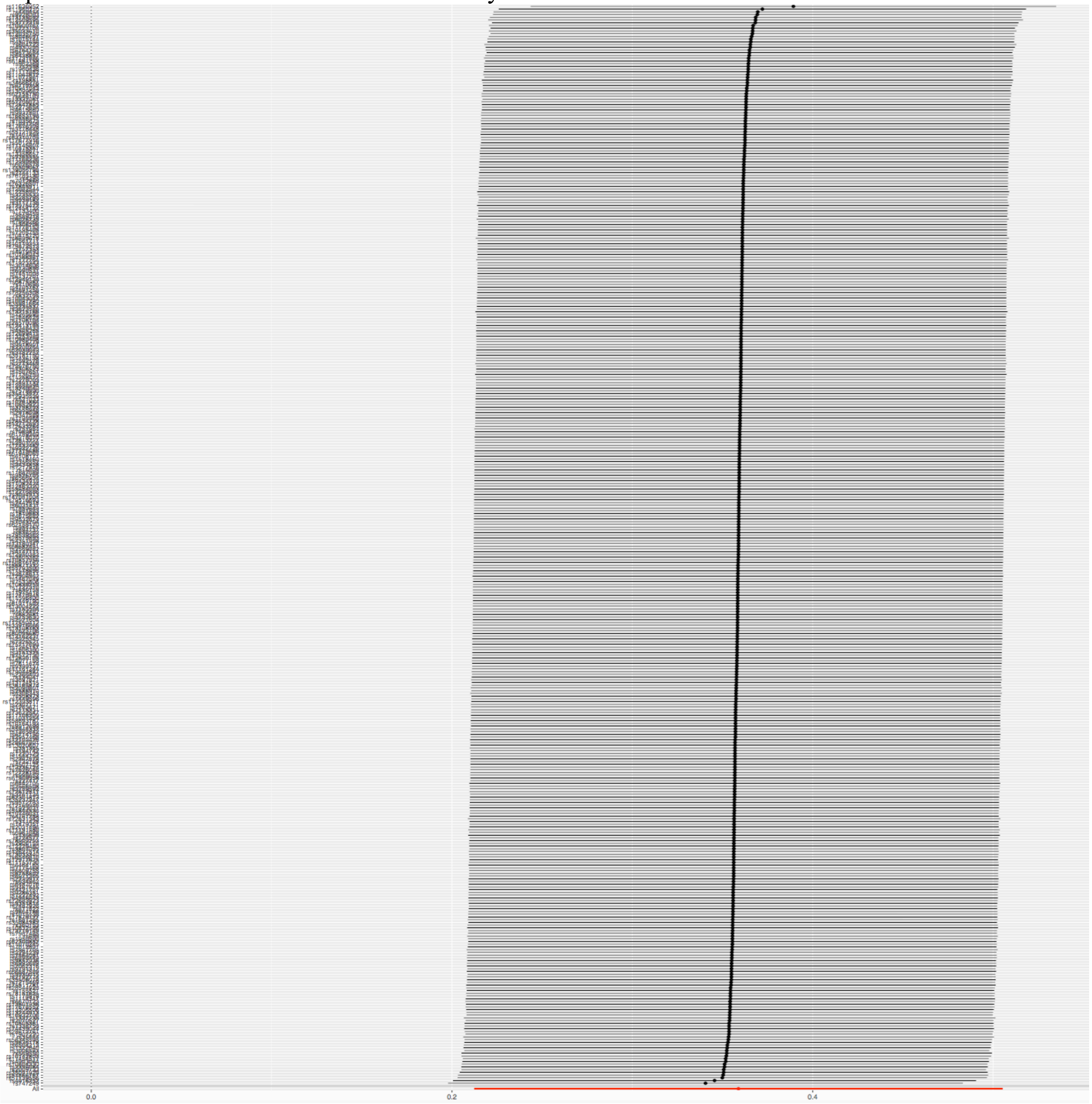


Supplementary Figure VIII - A diagnostic leave-one-out plot was generated for the diastolic blood pressure exposure Mendelian randomization analysis with the AAA outcome. Minimal evidence of a single association driving the inverse-variance weighted result was observed.

**Supplementary Figure IX -** Diagnostic funnel plot for diastolic blood pressure exposure-AAA Mendelian randomization analysis

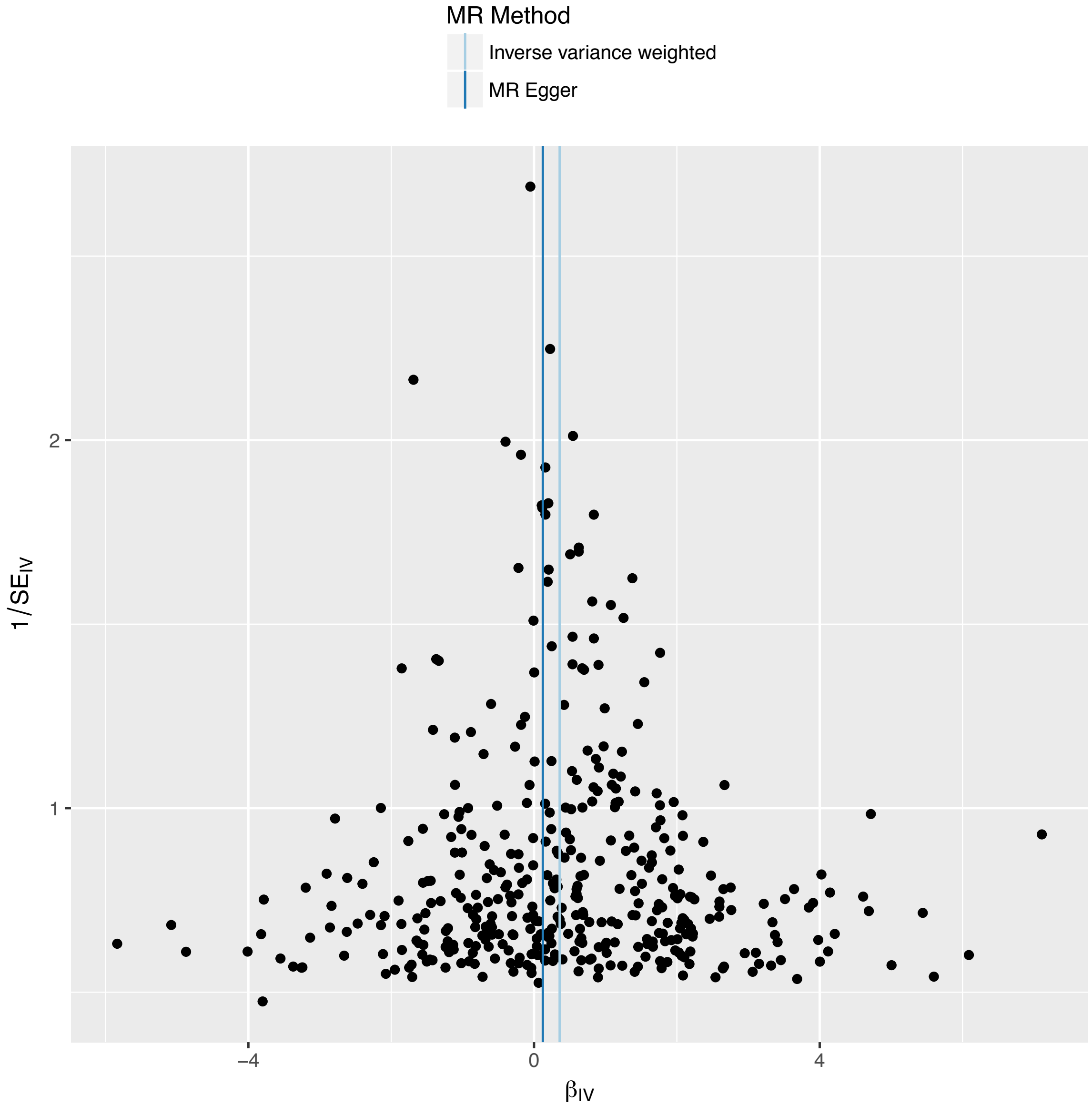


Supplementary Figure IX - A funnel plot depicting the reciprocal of the standard error (Y-axis) of the instrumental variable estimate against the instrumental variable estimates (X-axis) for diastolic blood pressure. Ideally, these plots should demonstrate a symmetric funnel, in which more precise estimates are less variable. Minimal asymmetry was observed.

Abbreviations: MR, Mendelian Randomization; IV, Instrumental Variable

**Legends for Supplementary Excel File Tables**

**Supplementary Table I** - Odds Ratios and P values for 10 previously identified genome-wide significant AAA loci in MVP discovery GWAS analysis (N = 7,642 AAA cases and 172,172 controls)

**Supplementary Table II -** Logistic regression odds ratios and two-sided P values for 14 novel genome-wide significant AAA loci in MVP discovery and replication analyses

**Supplementary Table III** - Logistic regression odds ratios and two-sided P values for suggestive loci in MVP discovery that did not meet prespecified criteria for replication (N = up to 12,614 cases and 272,030 controls - MVP+Stage2a; N = up to 10,477 cases and 279,641 controls - MVP+Stage2b)

**Supplementary Table IV** - Logistic regression phenome-wide association results (PheWAS) for AAA risk DNA sequence variants surviving Bonferroni Correction (two-sided P < 1.7E-6). All associations are aligned to the AAA risk allele

**Supplementary Table V** - LD Score regression genetic correlation ($r_g$) results for AAA and its risk factors (and risk factors for rupture). AAA summary statistics from MVP (N = 7,642 AAA cases and 172,172 controls) and the data source provided below were used for this analysis. Two-sided values of P are displayed

**Supplementary Table VI -** Logistic (smoking initiation, smoking cessation) and linear (smoking heaviness) regression effect estimates, standard errors, and two-sided P values of variants used for Mendelian randomization analysis. Effect estimates/P values are taken from 2019 GSCAN GWAS analysis summary-level data from up to 1,232,091 participants

**Supplementary Table VII** - Logistic regression association effect estimates, standard errors, and two-sided P values for the smoking phenotype-AAA Mendelian randomization analyses

**Supplementary Table VIII -** Linear regression SBP and DBP effect estimates, standard errors, and two-sided P values of variants used for Mendelian randomization analysis. Effect estimates/P values are taken from 2019 ICBP+UK Biobank discovery GWAS analysis summary statistics in up to 757,601 participants

**Supplementary Table IX** - Logistic regression association effect estimates, standard errors, and two-sided P values for the blood pressure-AAA Mendelian randomization analyses. Effect estimates are scaled to reflect odds of outcome per 10mmHg increase in each blood pressure trait.

**Supplementary Table X** - Logistic regression association results for AAA risk DNA sequence variants with cerebral, lower extremity, and iliac artery aneurysms. All

associations are aligned to the AAA risk allele and two-sided values of P are displayed. Nominally significant associations (P< 0.05) are highlighted in blue

**Supplementary Table XI** - Logistic regression association results for AAA risk DNA sequence variants with cerebral, lower extremity, and iliac artery aneurysms after excluding AAA cases. All associations are aligned to the AAA risk allele and two-sided values of P are displayed. Associations significant (P< 0.05) after excluding AAA cases from the analysis are highlighted in blue. A greater attenuation of association signal for the AAA risk variants with iliac artery and lower extremity aneurysms than for cerebral aneurysms was observed.

**Supplementary Table XII** - Overlap of diagnoses between AAA and lower extremity aneurysms/iliac artery aneurysms/cerebral aneurysms in MVP 2.1 data

**Supplementary Table XIII -** Variants used for 3 different weighted AAA polygenic risk scores ($PRS_{AAA}$), generated from MVP AAA summary statistics (N = 7,642 AAA cases and 172,172 controls)

**Supplementary Table XIV** - Demographic and clinical characteristics of Mayo Clinic Vascular Disease Biorepository AAA ascertained case-control cohort. All participants are of European ancestry

**Supplementary Table XV** - Baseline characteristics and AAA prevalence across MVP release 3.0 European and African ancestry individuals, BioMe, and the Penn Medicine Biobank

**Supplementary Table XVI** - Prevalence of AAA among the individuals in the top 5% $PRS_{AAA}$ in MVP (African and European ancestries), as well as Penn Medicine Biobank and BioMe (European ancestry) stratified by study, age, and gender

**Supplementary Table XVII** - Prevalence of AAA MVP release 3.0 (African and European ancestries), as well as Penn Medicine Biobank and BioMe (European ancestry) stratified by PRS quintile

## VA Million Veteran Program

### MVP Executive Committee

- Co-Chair: J. Michael Gaziano, M.D., M.P.H.
- Co-Chair: Rachel Ramoni, D.M.D., Sc.D.
- Jean Beckham, Ph.D.
- Jim Breeling, M.D. (ex-officio)
- Kyong-Mi Chang, M.D.
- Grant Huang, Ph.D. (ex-officio)
- Sumitra Muralidhar, Ph.D.
- Christopher J. O'Donnell, M.D., M.P.H.
- JP Casas Romero, M.D., Ph.D., Ex-Officio
- Philip S. Tsao, Ph.D.

### MVP Program Office

- Sumitra Muralidhar, Ph.D.
- Jennifer Moser, Ph.D.

### MVP Recruitment/Enrollment

- Recruitment/Enrollment Director/Deputy Director, Boston – Stacey B. Whitbourne, Ph.D.; Jessica V. Brewer, M.P.H.
- MVP Coordinating Centers
    - Clinical Epidemiology Research Center (CERC), West Haven – John Concato, M.D., M.P.H.
    - Cooperative Studies Program Clinical Research Pharmacy Coordinating Center, Albuquerque - Stuart Warren, J.D., Pharm D.; Dean P. Argyres, M.S.
    - Genomics Coordinating Center, Palo Alto – Philip S. Tsao, Ph.D.
    - Massachusetts Veterans Epidemiology Research Information Center (MAVERIC), Boston - J. Michael Gaziano, M.D., M.P.H.
    - MVP Information Center, Canandaigua – Brady Stephens, M.S.
- Core Biorepository, Boston – Mary T. Brophy M.D., M.P.H.; Donald E. Humphries, Ph.D.
- MVP Informatics, Boston – Nhan Do, M.D.; Shahpoor Shayan
- Data Operations/Analytics, Boston – Xuan-Mai T. Nguyen, Ph.D.

### MVP Science

- Genomics - Christopher J. O'Donnell, M.D., M.P.H.; Saiju Pyarajan Ph.D.; Philip S. Tsao, Ph.D.
- Phenomics - Kelly Cho, M.P.H, Ph.D.
- Data and Computational Sciences – Saiju Pyarajan, Ph.D.
- Statistical Genetics – Elizabeth Hauser, Ph.D.; Yan Sun, Ph.D.; Hongyu Zhao, Ph.D.

### MVP Local Site Investigators

- Atlanta VA Medical Center (Peter Wilson)

1670 Clairmont Rd, Decatur, GA 30033

- Bay Pines VA Healthcare System (Rachel McArdle)
  10,000 Bay Pines Blvd Bay Pines FL 33744
- Birmingham VA Medical Center (Louis Dellitalia)
  700 S. 19th Street Birmingham AL 35233
- Cincinnati VA Medical Center (John Harley)
  3200 Vine Street, Cincinnati, OH 45220
- Clement J. Zablocki VA Medical Center (Jeffrey Whittle)
  5000 West National Avenue, Milwaukee, WI 53295
- Durham VA Medical Center (Jean Beckham)
  508 Fulton Street Durham, NC 27705
- Edith Nourse Rogers Memorial Veterans Hospital (John Wells)
  200 Springs Road, Bedford, MA 01730
- Edward Hines, Jr. VA Medical Center (Salvador Gutierrez)
  5000 South 5th Avenue, Hines, IL 60141
- Fayetteville VA Medical Center (Gretchen Gibson)
  1100 N College Ave, Fayetteville, AR 72703
- VA Health Care Upstate New York (Laurence Kaminsky)
  113 Holland Avenue Albany NY 12208
- New Mexico VA Health Care System (Gerardo Villareal)
  1501 San Pedro Drive, S.E.Albuquerque, NM 87108
- VA Boston Healthcare System (Scott Kinlay)
  150 S. Huntington Avenue, Boston, MA 02130
- VA Western New York Healthcare System (Junzhe Xu)
  3495 Bailey Avenue Buffalo, NY 14215-1199
- Ralph H. Johnson VA Medical Center (Mark Hamner)
  109 Bee Street, Mental Health Research, Charleston, SC 29401
- Wm. Jennings Bryan Dorn VA Medical Center (Kathlyn Sue Haddock)
  6439 Garners Ferry Road, Columbia, SC 29209
- VA North Texas Health Care System (Sujata Bhushan)
  4500 S. LANCASTER ROAD, DALLAS, TX 75216
- Hampton VA Medical Center (Pran Iruvanti)
  100 Emancipation Drive, Hampton, VA 23667
- Hunter Holmes McGuire VA Medical Center (Michael Godschalk)
  1201 Broad Rock Blvd., Richmond, VA 23249
- Iowa City VA Health Care System (Zuhair Ballas)
  601 Highway 6 West, Iowa City, IA 52246-2208

- Jack C. Montgomery VA Medical Center (Malcolm Buford)
  1011 Honor Heights Dr., Muskogee, OK 74401
- James A. Haley Veterans' Hospital (Stephen Mastorides)
  13000 Bruce B. Downs Blvd., Tampa, FL 33612
- Louisville VA Medical Center (Jon Klein)
  800 Zorn Avenue, Louisville, KY 40206
- Manchester VA Medical Center (Nora Ratcliffe)

718 Smyth Road, Manchester, NH 03104

- Miami VA Health Care System (Hermes Florez)
  1201 NW 16th Street, 11 GRC, Miami FL 33125
- Michael E. DeBakey VA Medical Center (Alan Swann)
  2002 Holcombe Blvd. Houston TX 77030
- Minneapolis VA Health Care System (Maureen Murdoch)
  One Veterans Drive Minneapolis MN 55417
- N. FL/S. GA Veterans Health System (Peruvemba Sriram)
  1601 SW Archer Road, Gainesville, FL 32608
- Northport VA Medical Center (Shing Shing Yeh)
  79 Middleville Road, Northport, NY 11768
- Overton Brooks VA Medical Center (Ronald Washburn)
  510 East Stoner Ave, Shreveport, LA 71101
- Philadelphia VA Medical Center (Darshana Jhala)
  3900 Woodland Avenue, Philadelphia, PA 19104
- Phoenix VA Health Care System (Samuel Aguayo)
  650 E. Indian School Road, Phoenix, AZ 85012
- Portland VA Medical Center (David Cohen)
  3710 SW U.S. Veterans Hospital Road, Portland, OR 97239
- Providence VA Medical Center (Satish Sharma)
  830 Chalkstone Avenue, Providence, RI 02908
- Richard Roudebush VA Medical Center (John Callaghan)
  1481 West 10th Street, Indianapolis, IN 46202
- Salem VA Medical Center (Kris Ann Oursler)
  1970 Roanoke Blvd.,Salem, VA 24153
- San Francisco VA Health Care System (Mary Whooley)
  4150 Clement Street, San Francisco, CA 94121
- South Texas Veterans Health Care System (Sunil Ahuja)
  7400 Merton Minter Boulevard, San Antonio, TX 78229
- Southeast Louisiana Veterans Health Care System (Amparo Gutierrez)
  2400 Canal Street, New Orleans, LA 70119
- Southern Arizona VA Health Care System (Ronald Schifman)
  3601 S 6th Ave, Tucson, AZ 85723
- Sioux Falls VA Health Care System (Jennifer Greco)
  2501 W 22nd St, Sioux Falls, SD 57105
- St. Louis VA Health Care System (Michael Rauchman)
  915 North Grand Blvd., St. Louis, MO 63106
- Syracuse VA Medical Center (Richard Servatius)
  800 Irving Avenue, Syracuse, NY 13210
- VA Eastern Kansas Health Care System (Mary Oehlert)
  4101 S 4th Street Trafficway, Leavenworth, KS 66048
- VA Greater Los Angeles Health Care System (Agnes Wallbom)
  11301 Wilshire Blvd Los Angeles, CA 90073
- VA Loma Linda Healthcare System (Ronald Fernando)
  11201 Benton Street, Loma Linda, CA 92357

- VA Long Beach Healthcare System (Timothy Morgan)
  5901 East 7th Street   Long Beach CA 90822
- VA Maine Healthcare System (Todd Stapley)
  1 VA Center, Augusta, ME 04330
- VA New York Harbor Healthcare System (Scott Sherman)
  423 East 23rd Street   New York, NY 10010
- VA Pacific Islands Health Care System (Gwenevere Anderson)
  459 Patterson Rd, Honolulu, HI 96819
- VA Palo Alto Health Care System (Philip Tsao)
  3801 Miranda Avenue   Palo Alto, CA 94304-1290
- VA Pittsburgh Health Care System (Elif Sonel)
  University Drive, Pittsburgh, PA 15240
- VA Puget Sound Health Care System (Edward Boyko)
  1660 S. Columbian Way Seattle, WA 98108-1597
- VA Salt Lake City Health Care System (Laurence Meyer)
  500 Foothill Drive Salt Lake City, UT 84148
- VA San Diego Healthcare System (Samir Gupta)
  3350 La Jolla Village Drive, San Diego, CA 92161
- VA Southern Nevada Healthcare System (Joseph Fayad)
  6900 North Pecos Road, North Las Vegas, NV 89086
- VA Tennessee Valley Healthcare System (Adriana Hung)
  1310 24th Ave. South Nashville, TN 37212
- Washington DC VA Medical Center (Jack Lichy)
  50 Irving St, Washington, D. C. 20422
- W.G. (Bill) Hefner VA Medical Center (Robin Hurley)
  1601 Brenner Ave, Salisbury, NC 28144
- White River Junction VA Medical Center (Brooks Robey)
  163 Veterans Drive, White River Junction, VT 05009
- William S. Middleton Memorial Veterans Hospital (Robert Striker)
  2500 Overlook Terrace, Madison, WI 53705
